# Functional surface expression of immunoglobulin cleavage systems in a candidate *Mycoplasma* vaccine chassis

**DOI:** 10.1038/s42003-024-06497-8

**Published:** 2024-06-28

**Authors:** Sergi Torres-Puig, Silvia Crespo-Pomar, Hatice Akarsu, Thatcha Yimthin, Valentina Cippà, Thomas Démoulins, Horst Posthaus, Nicolas Ruggli, Peter Kuhnert, Fabien Labroussaa, Jörg Jores

**Affiliations:** 1 Institute of Veterinary Bacteriology, Department of Infectious Diseases and Pathobiology, Vetsuisse Faculty, University of Bern, 3001 Bern, Switzerland; 2 SIB Swiss Institute of Bioinformatics, Lausanne, Switzerland; 3 Institute of Animal Pathology, Department of Infectious Diseases and Pathobiology, Vetsuisse Faculty, University of Bern, 3001 Bern, Switzerland; 4 Department of Infectious Diseases and Pathobiology, Vetsuisse Faculty, University of Bern, 3001 Bern, Switzerland; 5 Institute of Virology and Immunology IVI, Sensemattstrasse 293, 3147 Mittelhäusern, Schweiz; 6 Multidisciplinary Center for Infectious Diseases (MCID), University of Bern, 3001 Bern, Switzerland; 7 French Agency for Food, Environmental and Occupational Health and Safety (ANSES), Lyon Laboratory, VetAgro Sup, UMR Animal Mycoplasmosis, University of Lyon, Lyon, France

**Keywords:** Bacteriology, Bacterial genes

## Abstract

The Mycoplasma Immunoglobulin Binding/Protease (MIB-MIP) system is a candidate ‘virulence factor present in multiple pathogenic species of the *Mollicutes*, including the fast-growing species *Mycoplasma feriruminatoris*. The MIB-MIP system cleaves the heavy chain of host immunoglobulins, hence affecting antigen-antibody interactions and potentially facilitating immune evasion. In this work, using -omics technologies and 5’RACE, we show that the four copies of the *M. feriruminatoris* MIB-MIP system have different expression levels and are transcribed as operons controlled by four different promoters. Individual MIB-MIP gene pairs of *M. feriruminatoris* and other *Mollicutes* were introduced in an engineered *M. feriruminatoris* strain devoid of MIB-MIP genes and were tested for their functionality using newly developed *oriC*-based plasmids. The two proteins are functionally expressed at the surface of *M. feriruminatoris*, which confirms the possibility to display large membrane-associated proteins in this bacterium. However, functional expression of heterologous MIB-MIP systems introduced in this engineered strain from phylogenetically distant porcine *Mollicutes* like *Mesomycoplasma hyorhinis* or *Mesomycoplasma hyopneumoniae* could not be achieved. Finally, since *M. feriruminatoris* is a candidate for biomedical applications such as drug delivery, we confirmed its safety in vivo in domestic goats, which are the closest livestock relatives to its native host the Alpine ibex.

## Abstract (web summary)

A microbiology study on the creation of plasmids for the candidate vaccine chassis *Mycoplasma feriruminatoris* and its use to study anti-Ig systems. The study also confirms safety of *M. feriruminatoris* when used in domestic goats.

## Introduction

Bacteria of the class *Mollicutes* are characterized by the absence of a cell wall and numerous enzymatic pathways that were lost by reductive evolution from Gram-positive ancestors. As a result, *Mollicutes* have a pleomorphic cell shape and live a parasitic lifestyle to scavenge nutrients from their host. A number of *Mollicutes* infecting plants or animals (including humans) are pathogenic, such as the well-known pathogens *Candidatus Phytoplasma asteris*^1^, members of the “*Mycoplasma mycoides* cluster”^2^ and *Mycoplasmoides* (*Mycoplasma*) *pneumoniae* and *Mycoplasmoides* (*Mycoplasma*) *genitalium*^3,4^, respectively. Knowledge of their virulence traits is still scarce due to the fastidious nature of these organisms and the historical lack of genetic tools to modify their genomes. Recently, the candidate virulence factor Mycoplasma Immunoglobulin Binding/Protease (MIB-MIP) system has been characterized in various *Mollicutes* species^5–8^. This system consists of at least two surface located proteins that bind and cleave the variable region of the heavy chain (V_H_) of IgGs and has been shown to be active in vivo in goats infected with *Mycoplasma mycoides* subsp. *capri* (*Mmc*)^9^. The structure of the two proteins in complex with an antibody was solved using cryo-electron microscopy. The two proteins bind to the Fab fragment in a “hug of death” mechanism, which is thought to interfere with antibody-antigen interactions^10^.

*Mycoplasma feriruminatoris* (*Mferi*) is a close relative of the *Mollicutes* belonging to the “*M. mycoides* cluster” and has been isolated from Alpine ibex and Rocky Mountain goats^11,12^. This species is characterized by its short doubling time compared to the slow growth typically observed in many other *Mollicutes*. The genome of *Mferi* has been recently adapted to synthetic genomics techniques^13^, including genome editing in *Saccharomyces cerevisiae* and genome transplantation^14^. All these features together with the simplistic nature of *Mollicutes*, absence of a cell wall and different genetic code have turned *Mferi* and other *Mollicutes* as promising candidates to serve as a workhorse for industrial applications^15^, such as in vivo vaccine or drug delivery vessel^13,16,17^.

In this work we analyzed in depth the operons expressing the four MIB-MIP gene copies present in *Mferi* by using -omics technologies and cDNA amplification. We also developed *oriC*-based plasmids to allow rapid introduction of genes and *in cellulo* expression of homologous and heterologous DNA at a high turnaround time. Using these tools, we could express functional MIB-MIP gene tandems individually to test their activity and we also attempted expression of heterologous MIB-MIP systems from other *Mollicutes* species, proving that *Mferi* is a valuable bacterial platform for functional genomics studies of this and other species. Finally, we also tested the pathogenicity of *Mferi* in domestic goats in vivo and demonstrated its safety, paving the way for future industrial applications.

## Materials and methods

All methods employed in this work that required use of commercial kits were performed following the manufacturer’s instructions, unless stated otherwise.

All primers used in this work for the different applications can be found listed in Supplementary Table S1.

### Strains used and culture conditions

*Escherichia coli* Stellar cells (Clontech) or NEB 5-alpha (New England Biolabs) were used for all constructions of different *oriC*-plasmids and subsequent plasmid preparations. All *E. coli* strains were cultured in Luria Bertani (LB) medium at 37 °C and shaking at 220 rpm or on LB agar plates supplemented with 100 µg mL^−1^ ampicillin when necessary. Transformation of *E. coli* strains was achieved by using a heat-shock standard protocol^18^.

*S. cerevisiae* strain W303a was used to modify and propagate the *Mferi* genome. *S. cerevisiae* was cultured in Yeast Peptone Dextrose Adenine or Synthetic Defined broth (Formedium) depleted for tryptophan, uracil and/or histidine depending on the auxotrophic marker in use. *S. cerevisiae* strains were cultured at 30 °C and 220 rpm.

*Mferi* IVB14/OD_0535^15^, *Mesomycoplasma* (*Mycoplasma*) *hyorhinis* JF5820^19^, and *Mesomycoplasma* (*Mycoplasma*) *hyopneumoniae* Ue273 used in this study were isolated from diagnostic material at the Institute of Veterinary Bacteriology in Bern. *M. hyopneumoniae* Ue273 was isolated from bronchial tissue of a Swiss wild boar. *Mferi* and *Mmc* strains used for -omics and in vitro studies were grown at 37 °C with 5% CO_2_ in SP5 medium^20^ or modified Hayflick agar plates^21^ supplemented with 15 µg mL^−1^ tetracycline or 16 µg mL^−1^ puromycin when necessary. Presence of *oriC*-plasmids in liquid cultures was maintained by puromycin (8 µg mL^−1^). *M. hyorhinis* and *M. hyopneumoniae* were grown in Friis medium^22^ at 37 °C. *Mycoplasma capricolum* subsp. *capricolum* ΔRE (*Mcap* ΔRE) was used as a recipient for genome transplantation from yeast^23^ (see “Genome transplantation” section below). For the experimental infection *M. capricolum* subsp. *capripneumoniae* (*Mccp*) ILRI181 and *Mferi* G5847^T^ were grown at 37 °C with 5% CO_2_ in Mycoplasma Experience Liquid Medium (Mycoplasma Experience), aliquoted and stored at −80 °C until used.

### Phylogenetic analysis

The phylogenetic analysis based on the 16S rRNA gene sequences of the *Mollicutes* covered overall *n* = 7 genera, encompassing *n* = 27 species and *n* = 36 strains. The tree was built in BioNumerics v8.1 using Jukes-Cantor correction and the Neighbour Joining method. *Clostridium innocuum* was used as outgroup.

For the phylogenetic analysis of the MIB-MIP system, the translated amino acid sequences were used using the genomes described above (Supplementary Data 1). Orphan copies of either the MIB or MIP were not included in the analysis. MIB and MIP protein sequences were first aligned separately with MAFFT v7.5^24^, then the alignments were concatenated with a custom R script. Phylogenetic reconstruction of the unrooted tree was performed with IQ-TREE 2.0.3^25,26^ for Linux, running with phyml parameter and 1000 replicates for both ultrafast bootstrap^27^ and likelihood ratio test. ModelFinder^28^ gave the LG+F+I+G4 model as best-fit. The tree was then plotted and manually edited in FigTree (v1.4.4).

### Genomic DNA extraction and Next Generation Sequencing

Genomic DNA (gDNA) from *Mollicutes* was extracted from 20 mL cultures using the Promega Wizard Genomic DNA purification kit. The quality and quantity of the gDNA was assessed on agarose gels and using the Qubit fluorometer (Invitrogen). Subsequently, gDNA was sequenced in the PacBio sequencing platform at the Lausanne Genomic Technologies Facility at the Center for Integrative Genomics (University of Lausanne), as described elsewhere^29^. High molecular weight DNA was sheared with Megaruptor (Diagenode, Denville, NJ, USA) to obtain 10 kb fragments. After shearing the DNA size distribution was checked on a Fragment Analyzer (Advanced Analytical Technologies, Ames, IA, USA). 500 ng of each DNA was used to prepare a SMRTbell library with the PacBio SMRTbell Express Template Prep Kit 2.0 (Pacific Biosciences). The resulting libraries were pooled with other libraries processed the same. The pool was size selected with Ampure PacBio beads to eliminate fragments <5 kb. It was sequenced with v3.2/v2.0 chemistry and diffusion loading on a PacBio Sequel II instrument (Pacific Biosciences) at 900 min movie length and pre-extension time of 120 min using one SMRT cell 8M. Demultiplexing, microbial assemblies and m6A-m4C base modification detection were performed using smrtlink v11.1. Genomes were assembled from PacBio reads with Unicycler v0.4^30^, smrtlink v11 or Flye v2.8^31^. Circularized genomes were polished with three rounds with the Arrow software [single-molecule real-time (SMRT) Link version 8 package]. Genomes were rotated to the first nucleotide of the start codon of the *dnaA* gene, and annotated using Prokka, version 1.13^31^. *M. hyopneumoniae* Ue273, *Mesomycoplasma* (*Mycoplasma*) *ovipneumoniae* 14KM848 and *Mferi* ΔMIB-MIP sequences are deposited as BioProject PRJNA1062711.

### Transcriptomic analysis and 5’ Rapid Amplification of cDNA Ends

RNA extraction for transcriptomics analysis was carried out as previously described^29^. Briefly, RNA from three biological replicates of *Mferi* was extracted from 5 mL liquid cultures using the Zymo Research Quick-RNA Fungal/Bacterial Miniprep kit. RNA quality was assessed at the Lausanne sequencing platform on a Fragment Analyzer (Agilent Technologies). Libraries were prepared using the Illumina TruSeq Stranded mRNA reagents (Illumina), excluding the polyA selection step and using a unique dual indexing strategy. Ribosomal rRNA depletion was carried out with QIAseq FastSelect–5S/16S/23S kit (Qiagen). Libraries were quantified by Qubit (Life Technologies) and their quality was assessed on a Fragment Analyzer (Agilent Technologies). Cluster generation was performed with 1.92 nM of an equimolar pool from the resulting libraries using the Illumina HiSeq 3000/4000 SR Cluster Kit reagents and sequenced on the Illumina HiSeq 4000 using HiSeq 3000/4000 SBS Kit reagents for 2 × 150 cycles (paired end). Sequencing data were demultiplexed using the bcl2fastq2 Conversion Software v. 2.20 (Illumina). Reads were first trimmed with fastp (0.19.5) and aligned against the reference genome (LR739236.1) with bwa mem (0.7.13). The mapped reads were filtered for quality, duplication and pairing with picard-tools (2.9.0) (http://broadinstitute.github.io/picard) and samtools (1.10)^32^. RNAseq coverage analysis was performed using defaults parameters from geneBody_coverage and FPKM_count from the RSeQC (v5) package^33^. Then resulting coverage and histograms plots were done in R.

5’ Rapid Amplification of cDNA Ends (5’RACE) was performed using the 5’/3’RACE kit (Roche), except for the PCR amplification steps, in which dA-tailed cDNA was amplified using Q5 polymerase (New England Biolabs) instead. The PCR conditions were the following: initial denaturation at 98 °C for 2 min; 10 cycles of 15 s at 98 °C, 30 s at 54 °C and 40 s at 72 °C; 10 cycles of 15 s at 98 °C, 30 s at 54 °C and 90 s at 72 °C; 15 cycles of 15 s at 98 °C, 30 s at 54 °C and 180 s at 72 °C; and a final extension of 7 min at 72 °C. The resulting PCR products were diluted 20x in distilled water and 1 µL was used for the subsequent nested PCR, using the same PCR protocol except that the annealing temperature in all cycles was increased to 56 °C. Final PCR products were purified using High Pure PCR product Purification Kit (Roche) and visualized in a 2% agarose gel. Purified PCR products were Sanger sequenced using the gene specific primers (Microsynth) and also assembled into pUC57mini vectors (Genscript) using the NEBuilder Hi-Fi DNA Assembly kit (NEB) prior transformation into *E. coli* DH5α. Several clones resulting from transformation were further screened by PCR and Sanger sequencing (Microsynth).

### Proteomics analyses

Proteomics analyses were performed as previously described^29^. Briefly, the same three biological replicates of mycoplasmas used for transcriptomics were harvested by centrifugation at 4000 × *g* at 4 °C for 15 min. Pellets were washed three times in ice-cold PBS and stored at −80 °C until further use. Cell pellets were reconstituted in 500 µL 8 M urea, 100 mM Tris/HCl pH 8, protein concentration determined with Pierce BCA Protein Assay Kit (ThermoScientific), and cysteines alkylated with 50 mM iodoacetamide for 30 min in the dark after reduction with 10 mM DTT for 30 min at 37 °C^34^. Proteins were precipitated with five volumes of cold acetone over night at −20 °C. Proteins were dissolved in 8 M urea, 50 mM Tris/HCl pH 8 at a concentration of 1 mg mL^−1^. An aliquot of 10 µL was further processed by lowering the urea concentration to 1.6 M with 20 mM Tris/HCl pH 8 containing 2 mM CaCl_2_, followed by digestion with 200 ng trypsin over night at room temperature. After acidification with 1% trifluoroacetic acid end concentration, aliquots of 5 µL (500 ng protein digest) were analyzed by nano-liquid reversed phase chromatography coupled to tandem mass spectrometry on an Orbitrap Fusion LUMOS mass spectrometer that was coupled with a Dionex Ultimate 3000 nano-UPLC system (ThermoFischer Scientific). A standard data-dependent acquisition method as described elsewhere^35^ was used with a homemade AcquityTM CSH C18 separation column (1.7 µm, 130 Å, 75 µm × 20 cm) at a flow rate of 250 nL min^−1^.

Mass spectrometry-derived proteomic data were analyzed against LR739236.1 Genbank file with concatenated reverse sequence decoys by Transproteomic pipeline (TPP) tools^36^. The five database search engines Comet^37^, Xtandem^38^, MSFragger^39^, MS-GF+^40^, and MyriMatch^41^ were used and each search was followed by the application of the PeptideProphet tool^42^. The iProphet^43^ software was subsequently used to summarize the search results, which were filtered at the false discovery rate of 0.01; furthermore, identifications were exclusively accepted if at least three of the search engines agreed on the identification. Protein inference was performed using ProteinProphet. For those protein groups accepted by a false discovery rate filter of 0.01, a Normalized Spectral Abundance Factor (NSAF)^44^ was calculated based on the peptide to spectrum match count. Shared peptides were considered by a method published elsewhere^45^. The mass spectrometry proteomics data have been deposited to the ProteomeXchange Consortium via the PRIDE^46^ partner repository with the dataset identifier PXD053286.

### CReasPy-Cloning of *M. feriruminatoris* genome

A mutant strain of *Mferi* IVB14/OD_0535 without the four MIB-MIB gene pairs (ΔMIB-MIP) was generated using the CReasPy-Cloning method^47^. Briefly, two different gRNA sequences were designed by using the “CRISPR Guides” tool available in the Benchling work environment (https://benchling.com). Target sequences with the highest on-target score and the lowest off-target score were selected. Two gRNAs were designed to target the genes coding for the 4 copies of the MIB-MIP system in *Mferi* (locus_tags MF5583_00301 - MF5583_00308), by using complementary primers (#084-087). The corresponding pgRNA plasmids (pgRNA-1MIBMIP, pgRNA-2MIBMIP) were constructed following the protocol described elsewhere^48^. Plasmids were sequence verified using Sanger sequencing (Microsynth) and purified using QIAprep Miniprep Spin Kit (Qiagen). Plasmids were then transformed into *S. cerevisiae* W303a-eSpCas9 via lithium acetate transformation^49^ and transformants were selected on SD-Trp-Ura medium (Takara). Recombination templates containing the yeast elements (CEN-ARS + HIS3) and the tetracycline resistance cassette (pS’*tetM*) were produced by PCR amplification of the ARS4/CEN6/HIS/pS’*tetM* loci from the plasmid pMT85–PSTetM-ARSCenHis-pRS313^20^ using primers #082 and #083 with the Q5 High-Fidelity DNA Polymerase (New England Biolabs) and purified using the High Pure PCR Product Purification Kit (Roche). The resulting recombination template contained sequences with 50 bp homology to the regions flanking the MIB-MIP locus of the *Mferi* genome. *Mferi* genomes were isolated and introduced in *S. cerevisiae* W303a-eSpCas9 pgRNA-1MIBMIP or pgRNA-2MIBMIP by spheroplast transformation^50^, as previously described^13^. Yeast Artificial Chromosomes (YACs) containing the modified *Mferi* genomes were isolated as previously described using the CHEF Mammalian Genomic DNA Plug kit (BioRad)^14^.

### Genome transplantation into *Mcap* ΔRE recipient cell

The genome IVB14/OD_0535::YCp-ΔMIB-MIP maintained in *S. cerevisiae* as a YAC was transplanted into *Mcap* ΔRE strain as previously described^13,23^. Briefly, *Mcap* ΔRE was grown in SOB+ medium until early stationary phase (pH 6.5), washed in 10 mM Tris 250 mM NaCl pH 6.5 and resuspended in cold 0.1 M CaCl_2_. Transplantation was carried out mixing SP5 without serum and agarose plugs containing the modified genome with the resuspended cells in 2X Fusion Buffer (20 mM Tris, 20 mM MgCl_2_, 500 mM NaCl, 10% PEG_6000_ pH 6.5). Mixtures were incubated statically for 90 min at 30 °C and then plated in SP5 plates supplemented with 15 µg mL^−1^ tetracycline. The resulting transplanted mutant strains were passaged three times in liquid selective medium and pre-screened by MALDI-TOF MS identification using a Microflex LT instrument (Brucker). Moreover, clones were also subjected to multiplex and simplex PCRs to confirm integrity of the genome in selected transplanted clones. Additionally, the genomes of mutant strains were verified by PacBio sequencing and mapping assembly to the *Mferi* IVB14/OD_0535 parental strain^15^. Full genome sequence can be found in BioProject PRJNA1062711.

### Growth rate determination

Growth rate of the different mycoplasma strains was assessed by color-changing units (CCU) per mL as well as colony-forming units (CFU) for up to 20 h every 60 min. Briefly, 100 mL SP5 at pH = 7.5 containing 10^2^ cells mL^−1^ were incubated at 37 °C, and 5% CO_2_ and small aliquots were removed every hour. Each aliquot was serially diluted and distributed in 200 µL volumes using 96-well plates (for CCU calculation) and plated as spot dilutions in SP5 agar plates (for CFU calculation). Color change and colonies were assessed after 2 days of incubation. To better compare the growth rate between wild-type (WT) and mutant strains, no additional antibiotics were added to the SP5 medium during the experiment. Growth curves were plotted, and the growth rates were calculated using GraphPad Prism v9.0.0

### Plasmid construction

All plasmids generated in this study are listed in Supplementary Table S2. All plasmids were constructed using the NEBuilder HiFi DNA Assembly kit (New England Biolabs). The plasmid pIVB03 was constructed by replacing the origin of replication of *Mmc* GM12 present in pMYCO1^51^ by the origin of replication of *Mferi* type strain G5847^T^^11^, amplified with primers #001 and #002. The plasmid pIVB04 is a derivate of pIVB03 in which the *tetM* marker under the control of the spiralin promoter (pS’) was replaced by pS’*pac* marker, amplified from the gDNA of *Mcap* ΔRE strain^23^ using primers #003 and #004. The plasmid pIVB06 is a derivate of pIVB04 in which the orientation of the pS’*pac* marker was switched. This switch was performed by amplifying the pIVB04 backbone without the pS’*pac* marker using primers #007 and #008 and the pS’*pac* marker using primers #005 and #006. Plasmid pIVB08 is a derivate of plasmid pIVB03 and was constructed by assembling the pS’*tetM* marker amplified with primers #011 and #012 with the pIVB03 backbone amplified in two parts using primers #009 with #010, and #013 with #014. The plasmid pIVB09 was built by replacing the pS’*tetM* cassette from pIVB08 by the pS’*pac* marker, employing primers #009 and #016 for the amplification of the pIVB08 backbone and primers #011 and #015 to amplify the pS’*pac* marker.

All plasmids carrying MIB-MIP copies of the different *Mollicutes* have the pIVB09 as a backbone, amplified using primers #017 and #018. The MIB-MIP gene pairs and their natural promoter regions were amplified from gDNA of each respective host strain (*Mferi* IVB14/OD_0535, *M. hyopneumoniae* Ue273, *M. hyorhinis* JF5820). To generate plasmids expressing MIB-MIP gene copies with the promoter region of the first MIB-MIP gene copy of *Mferi* IVB14/OD_0535 (P_MM1mfe_), the pIVB09 backbone containing the P_MM1mfe_ was amplified using primers #017 and #051 and each MIB-MIP copy with their respective primer set. Plasmid carrying the tagged MIB-MIP copy number 4 from *Mmc* was created amplifying the promoter sequence of the first MIB-MIP copy of *Mmc* using primers #057 and #058, and each gene with primers #059 and #060, and #061 and #062. Plasmids bearing codon-optimized MIB-MIP gene pairs tagged with 6xHis and FLAG, respectively, were created by cloning each gene copy, which was custom synthesized (GenScript). Optimization was carried out by using the Optimizer tool^52^, using the guided random method and the codon table of *M. mycoides* subsp. *capri* (species 40477). The plasmids carrying transcriptional fusions of the monomeric Kasubira-Orange2 (mKO2) gene with the promoter regions of MM2_mfe_ and MM3_mfe_ were built by assembling the mKO2 gene (custom synthesized by Genscript) amplified with primers #096/#097 and #098 with the promoter regions amplified with #021 + #094 and #023 + #095. The promoter-less version was created using primer #098 + #099.

All plasmids were sequence-verified by Sanger sequencing at Microsynth AG (Switzerland). Sequences of all plasmids can be found in the Supplementary Data 3.

### Transformation of mycoplasmas and screening of mutants

The transformation protocol used to transform *oriC*-plasmids into *Mferi* IVB14/OD_0535 was adapted from the one used for transformation of *Mmc*^23^, with some modifications. *Mferi* was grown overnight (O/N) in SP5 with pH adjusted to 8.0 (SP5_pH8_) until late logarithmic phase (~pH 7.0) to achieve the highest total CFU mL^−1^ (4 mL culture/ transformation). Cells were cooled on ice, pelleted at 4200 × *g* for 15 min at 4 °C and washed once in Sucrose/Tris Buffer (0.25 M Sucrose, 10 mM Tris-HCl, pH 7.0). Each cell pellet was resuspended in 400 µL cold CaCl_2_ 0.1 M and kept on ice for 30 min. In a 50 mL falcon tube, 10 µL of plasmid (600–1000 ng µL^−1^) were added to 400 µL Fusion Buffer 2X (0.5 M Sucrose, 20 mM Tris-HCl, 40% PEG8000, pH 7.0) and left at room temperature. After the incubation of 30 min on ice, the 400 µL cell suspension was added into the Falcon tube containing the Fusion Buffer 2X as well as the plasmid and mixed gently. The reaction was left incubating at 30 °C for 25–30 min. Then, fusion reaction was stopped by adding 9 mL of cold SP5 and inverting the tube once. Cells were recovered by centrifugation at 4200 × *g* for 15 min at 10 °C and the supernatant was carefully discarded. The cell pellet was resuspended in 1 mL fresh SP5_pH8_ and incubated at 37 °C for 45–60 min before plating on selective agar plates. Colonies of transformants were visible after 48 h but were picked up on day 3–4 after transformation in 1.5 mL microcentrifuge tubes containing 1 mL SP5_pH8_ with the appropriate antibiotic (i.e., 16 µg mL^−1^ puromycin or 15 µg mL^−1^ tetracycline). Transformants were passaged three times before screening. Correct transformants were verified by PCR of cell lysates obtained as previously described^20^.

### Assessment of plasmid stability

*Mferi* strain carrying the pIVB09 plasmid was grown overnight in SP5_pH8_ supplemented with 16 µg mL^−1^ of puromycin, serially diluted and plated in non-selective SP5 plates and SP5 plates containing 16 µg mL^−1^ of puromycin (Passage P_0_). Stability and plasmid retention was studied along 10 passages (1:1000 dilution) in SP5_pH8_ supplemented with two concentrations of puromycin (8 or 16 µg mL^−1^) or non-selective conditions. CFU mL^−1^ were counted for each passage in selective (16 µg mL^−1^ puromycin) and non-selective plates. Plasmid retention ratios were calculated by dividing CFU mL^−1^ obtained in selective plates by total CFU mL^−1^ in each passage.

### MIB-MIP-derived IgG cleavage analysis

IgG cleavage assays were performed as described in refs. ^5,10^, with some modifications. For all *Mferi* strains, cultures were grown O/N in SP5_pH8_ with antibiotic selection, if necessary, until stationary phase. Next day, 1 mL fresh SP5_pH8_ medium was inoculated with 50 µL of the O/N cultures maintaining the antibiotic selection when necessary and grown at 37 °C and 5% CO_2_ until late logarithmic phase (~10^9^ cells). Cells were spun down at 7000 × *g* for 10 min, washed once with SP5 w/o serum^20^, and spun down again in the same parameters. Pellets were resuspended in 35 µL of SP5 w/o serum containing 250 ng µL^−1^ of purified IgG from goat or swine serum (Sigma-Aldrich) and incubated at 37 °C for 45 min. Then, cells were pelleted at 7000 × *g* for 10 min, and supernatants were recovered and mixed with 7 µL 6× Laemmli buffer before boiling at 100 °C for 10 min. Supernatants were separated in a 10% SDS-PAGE and transferred to a PVDF membrane using a Trans-Blot Turbo Transfer System (BioRad). Membrane was blocked in PBS with 5% skimmed milk (Becton Dickinson) and 0.05% Tween-20 (Sigma). Antibodies and working dilutions are listed in Supplementary Table S3. Membrane was developed using SuperSignal West Pico PLUS Chemiluminiscent substrate (ThermoFisher Scientific). In the case of *M. hyorhinis* and *M. hyopneumoniae*, IgG cleavage determination was performed similarly, but cells were grown in Friis medium until early stationary phase and no subculture step was performed. Besides, incubation with IgG was extended to 2 h (*M. hyorhinis*) or 3 h (*M. hyopneumoniae*).

### Analysis of expression of MIB, MIP and mKO2

Expression of C-terminal tagged MIB and MIP, or mKO2 was analyzed by immunoblot using anti-6xHis, anti-FLAG or anti-mKO2 antibodies, respectively (Supplementary Table S3). 1 mL of culture was used for each total protein extraction. Bacterial cells were washed twice in sterile PBS before suspended in 100 µL of PBS. Lysates were obtained by adding 20 µL 6× Laemmli buffer and boiling at 100 °C for 10 min. Lysates were separated in a 7.5% or 15% SDS-PAGE before blotting. The immunoblots were performed as described above. For membrane protein enrichment by Triton X-114 fractioning, samples were treated according to protocols published elsewhere^53,54^.

### Fluorescence microscopy of *Mferi* colonies

*Mferi* cultures were diluted and seeded in puromycin selection plates and incubated at 37 °C 5% CO_2_ for 2 days. Then, plates were observed under a Nikon Eclipse Ts2-FL inverted microscope with a TRITC filter cube and a DS-Fi3 camera under the control of NIS-Elements software. To take the fluorescence pictures, colonies were exposed for 800 ms. Composed images were merged by using ImageJ software.

### Statistical analysis

All analyses were carried out using GraphPad Prism (v9.0.0). One-way ANOVA tests and Tukey’s comparative tests were performed to assess significance and calculate *p* values when indicated.

### In vivo infection of domestic goats

The experimental infection of goats was performed in compliance with the Swiss animal protection law (TSchG SR 455; TSchV SR 455.1; TVV SR 455.163) under the cantonal license BE67/19. The experiments were reviewed by the cantonal committee on animal experiments of the canton of Bern, (Switzerland) and approved by the cantonal veterinary authority (Amt für Landwirtschaft und Natur LANAT, Veterinärdienst VeD, Bern, Switzerland). We have complied with all relevant ethical regulations for animal use.

The infection trial was carried out at the Institute of Virology and Immunology (Mittelhäusern, Switzerland) using six female goats in total, aged between 1 and 4 years old and weighting 50–60 kg, and randomly split into two groups of three animals. Animals were infected intranasally with 1 mL 10^8^ CCU mL^−1^ of *Mferi* strain G5847^T^, or *Mccp* strain ILRI181 on two consecutive days as described recently ^55^. One mL of mycoplasma culture was atomized using a 1 mL syringe attached with a MAD Nasal^TM^ Intranasal Mucosal Atomization Device (Teleflex) and 500 µL of aerosol was applied to each nostril. At 4 days post-infection (dpi), each animal was infected transtracheally with the same number of color changing units (10^8^ CCU mL^−1^). One mL of culture was followed by flushing with 5 mL sterile phosphate buffered saline. For the duration of the experiment, the animals were housed in a high containment facility, one stable per group of infection. The animals were kept on straw with a local ambient temperature of 20–22˚ C. Before and after *Mferi* or *Mccp* infection, body temperature and clinical status was monitored daily. The clinical status was assessed by a veterinarian, always the same person to ensure unbiased clinical assessment. The rectal body temperature was measured with a digital thermometer, whereas respiratory and heart frequencies employed a stethoscope. Differential blood cell counts were determined from EDTA blood samples using an automated hematology analyzer (VetScan). Animals were euthanized when endpoint criteria were reached or at the end of the trial and subjected to postmortem analysis. Endpoint criteria were the same as reported elsewhere^55^.

### Reporting summary

Further information on research design is available in the Nature Portfolio Reporting Summary linked to this article.

## Results

### MIB-MIP tandem gene copies of *Mferi* IVB14/OD_0535 are closely related to the ones present in *Mmc*

**Fig. 1:**
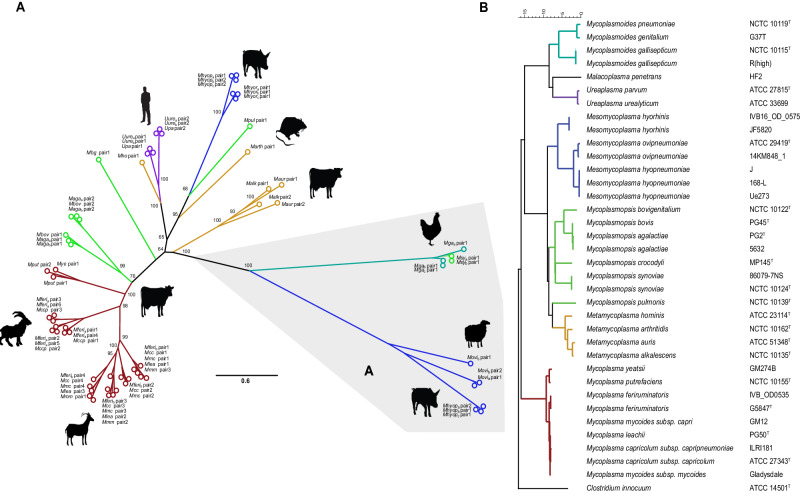
Phylogenetic analysis of paired MIB-MIP systems in different *Mollicutes* species. **A** Unrooted phylogenetic tree representing concatenated MIB-MIP protein pairs in representative strains of different species of *Mollicutes*. Colors indicate the different phylogenetic groups: *Mycoplasma* (in dark red), *Mycoplasmopsis* (in green), *Metamycoplasma* (in dark yellow), *Ureaplasma* (in purple), *Mesomycoplasma* (in blue) and *Mycoplasmoides* (in turquoise). Animal hosts of the different species are also displayed. The most distant branch is highlighted in gray. More information regarding strains and proteins displayed in the tree can be found in Supplementary Table S2. **B** Rooted phylogenetic tree showcasing the phylogenetic distance of representative strains of *Mycoplasmatota* according to the 16S RNA. *Clostridium innocuum* is displayed as an outgroup. Colors are used to distinguish between different phylogenetic groups as in (**A**).

The MIB-MIP system is widespread between different members of the *Mollicutes*^5^ and is a candidate virulence factor with potential implications in immune evasion^9,10^. *Mferi* strain G5847^T^ was shown to contain a large repeat of the MIB-MIP encoding region in the chromosome, leading to the presence of 2 identical sets of three MIB-MIP gene copies^13^. This unusual configuration is not present in any other isolate so far, which prompted us to study the occurrence and diversity of the MIB-MIP gene copies in *Mferi* IVB14/OD_0535 in respect to the rest of the *Mollicutes*. Therefore, we selected *Mollicutes* genomes, including the ones reported in this study, and searched for the presence of the MIB-MIP system. Altogether, we included a total of 34 genomes from 23 different species (Fig. 1A). We analyzed the sequences of 70 genes coding for putative MIB proteins and 71 genes encoding putative MIPs. In most genomes analyzed, both MIB and MIP counterparts were found adjacent in the same genetic locus, most likely forming a single transcriptomic unit. Occasionally we observed “orphan” MIB- or MIP-encoding genes. However, in these genomes another MIB-MIP pair was always present elsewhere in the genome, suggesting that Ig cleavage activity can potentially still occur. To facilitate the analysis, only the paired MIB-MIP proteins were included in the construction and analysis of the phylogenetic tree (Fig. 1A).

An initial scrutiny of the phylogenetic relations between MIB-MIP proteins of different species revealed three major distinctive branches, (i) encompassing *Mycoplasma* and *Mycoplasmopsis* species infecting ruminants; (ii) *Ureaplasma*, *Metamycoplasma* and some MIB-MIP pairs of porcine *Mesomycoplasma* species; and (iii) a more distant branch containing the MIB-MIP pairs of *Mycoplasmoides* and *Mesomycoplasma* species. We detected several examples of closely related MIB-MIP systems between *Mollicutes* species that are phylogenetically very distant but share the same hosts, such as *Ureaplasma* species and *Metamycoplasma hominis*, *Mycoplasmoides gallisepticum* and *Mycoplasmopsis synoviae*, or *Mycoplasmopsis pulmonis* and *Metamycoplasma arthritidis*, infecting humans, poultry, or rodents, respectively (Fig. 1A, B), as previously reported^5^. Here we found that there is a strong conservation between each pair in the same position, suggesting that multiple copies of this tandem system were present in a common ancestor of these mycoplasmas. Furthermore, there is a significant difference between the two strains of *Mferi* analyzed, as the MIB-MIP pairs of the type-strain G5847^T^ are similar to the ones present in *Mccp*, while the MIB-MIP pairs of the IVB14/OD_0535 strain cluster closely to the ones of the other members of the “*M. mycoides* cluster”. Remarkably, the two paired MIB-MIP copies of *M. hyopneumoniae*, located on different chromosomal loci, are highly divergent, with one MIB-MIP being more similar to the only system present in *M. hyorhinis* and the other more similar to MIB-MIP present in *Mesomycoplasma ovipneumoniae* (Fig. 1A). Overall, our analysis shows that the MIB-MIP system of *Mferi* might vary significantly between isolates, suggesting horizontal gene acquisition from different ancestors to different members of this species.

### Analysis of MIB-MIP expression in *Mferi*

**Fig. 2:**
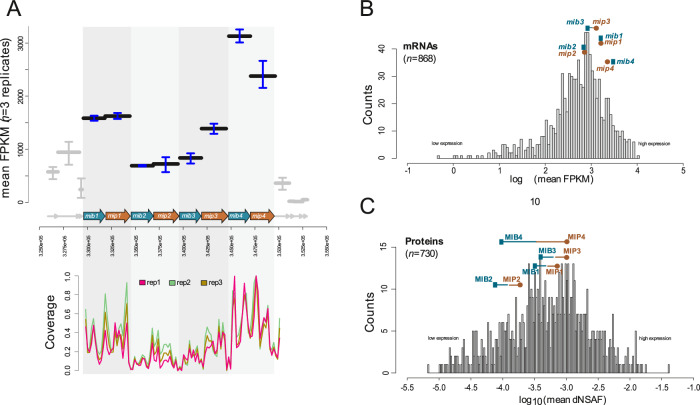
Analysis of MIB-MIP gene pair expression in *Mferi* using -omics. **A** Transcriptional analysis using RNAseq of three biological replicates. Top graph shows the mean fragments per kilobase of transcript per million mapped reads (FPKM) of each gene of the MIB-MIP cluster. Bottom graph indicates the read coverage obtained in each replicate. **B** Total gene distribution based on gene expression measured by RNAseq. **C** Total protein distribution based on protein expression measured by mass spectrometry.

We studied the expression of each MIB-MIP gene pair of this species by transcriptomics and proteomics analyses to see whether the gene pairs are organized as operons and to identify different promoters that can be used to express MIB-MIP pairs or other genes from other *Mollicutes* species. Exploration of the transcriptomic data showed that each MIB-MIP pair could constitute an individual transcriptional unit, with the first and last pairs (MM1_mfe_ and MM4_mfe_) being transcribed at higher levels than the other two pairs (MM2_mfe_ and MM3_mfe_) (Fig. 2A). Moreover, all MIB-MIP gene pairs are among the mid-to-high expressed genes of *Mferi*, with no big apparent differences between MIB and MIP transcription (Fig. 2B). However, when analyzing the proteomics data, MIB-MIP pairs are not among the highly expressed proteins of the cell highlighting the lack of correlation between RNA and protein levels (Fig. 2C). Besides, the protein expression levels of all MIP copies of each pair are significantly higher compared to their MIB counterparts. Given the similar disposition and number of the different MIB-MIP gene pairs between *Mferi* IVB14/OD_0535 and *Mmc* GM12, we also analyzed transcriptomics and proteomics data of GM12 obtained in a previous study^29^. Our results showed a similar mRNA expression trend for each MIB-MIP pair, with the first tandem of genes being transcribed higher than the rest (Supplementary Fig. S1). Moreover, proteomics data showed that most MIP proteins are expressed at higher levels than their respective MIB protein partner, with most of them being not reliably detected (Supplementary Fig. S1). Overall, these results suggest that each MIB-MIP system likely constitutes an individual expression unit and that expression of the different tandem of genes could be species-specific, despite having similar genetic organization or distribution in the different chromosomes.

### Identification of MIB-MIP promoter and terminator regions

**Fig. 3:**
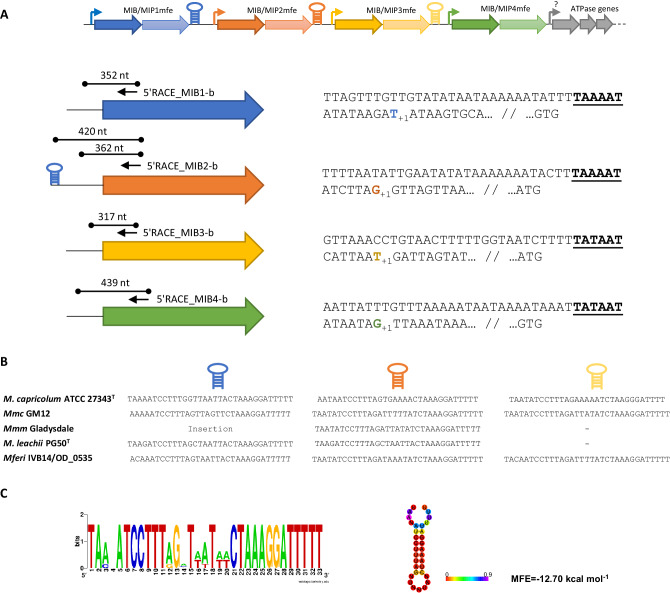
Analysis of the operonic structure of the MIB-MIP gene cluster. **A** Top section depicts the schematic organization of the chromosomal locus where the MIB-MIP gene cluster is located in *M. feriruminatoris* IVB14/OD_0535. Small arrows indicate promoter locations, while hairpins indicate the presence of putative terminator sequences. Bottom section illustrates the results obtained in the 5’RACE experiment. Arrows indicate the approximate location of the primers used in each gene. Length of each fragment obtained is also indicated. On the right, promoter sequences of each MIB copy are displayed. The transcriptional start site is indicated with a _+1,_ and the corresponding Pribnow box is highlighted in bold letters and underlined. The starting codon of each gene is shown at the end. **B** Presence of large, inverted repeats after the MIP genes of several species of the “*Mycoplasma mycoides* cluster”. First MIB-MIP operon of *Mmm* strain Gladysdale is interrupted by an insertion sequence. *Mmm* strain Gladysdale and *M. leachii* strain PG50 only have three MIB-MIP gene copies, therefore the last inverted repeat is absent. **C** Consensus sequence of the inverted repeat built with WebLogo tool. Sequence can form a hairpin loop with a minimum free energy (MFE) of −12.70 kcal/mol, as calculated with RNAfold webserver.

To further characterize the transcriptional profile shown by the RNAseq analysis of the MIB-MIP locus of *Mferi*, we decided to experimentally determine the transcriptional start sites (TSSs) present in that genomic area by 5’ Rapid Amplification of cDNA Ends (5’RACE). We could detect a single band for all MIB-MIP gene tandems, except for the second MIB-MIP pair in which two discreet bands were obtained (Fig. 3A and Supplementary Fig. S2). In this case, the smaller band could correspond to a TSS derived from an active promoter, while the larger product starts immediately after a large inverted repeat element. With these results, we could pinpoint the Pribnow box of each pair and no clear −35 element, which is quite common in mycoplasma species^56^. Moreover, genetic analysis of the downstream regions of MIB-MIP gene clusters in a number of members of the “*Mycoplasma mycoides* cluster” revealed the presence of similar inverted repeat elements with a structure reminiscent of rho-independent terminators (Fig. 3B). These elements are 32–34 bp long and are located very close to the stop codon of the MIP genes. The last MIB-MIP gene pair is always devoid of this downstream element in all studied species, suggesting that it is part of a larger operon unit that also comprises putative ATPase-coding genes located downstream of the MIB-MIP cluster. The consensus sequence of these elements was determined as 5’TA(A/C)NATCCTTT(A/G)G-NT(A/T_2_)T(A/T_2_)-CTAAAGGATTTTT using all available sequences (Fig. 3C). Employing RNAfold^57^, the RNA structure of these downstream elements was predicted to be a small hairpin with a minimum free energy of approximately −12.70 kcal mol^−1^ (Fig. 3C).

### Generation and characterization of a *M. feriruminatoris* ΔMIB-MIP strain

**Fig. 4:**
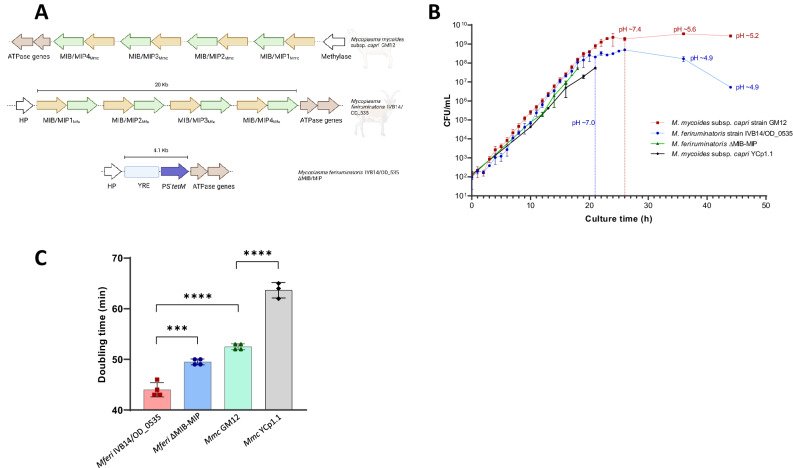
Construction and characterization of a MIB-MIP deficient *Mferi* strain. **A** Schematic representation of the MIB-MIP operons and their genomic context in the ruminant mycoplasmas *Mmc* GM12 and *Mferi* IVB14/OD_0535. Deletion of the four MIB-MIP gene pairs of *Mferi* results in the ΔMIB-MIP strain, in which these genes (from the start codon of the first gene to the stop codon of the last gene, ~20 Kb) have been replaced by a tetracycline resistance cassette (PS’*tetM*) and yeast replication elements (YRE) (~4.1 Kb). **B** Growth curve determined by CFU mL^−1^ of at least three independent biological replicates of cultivated *Mmc* GM12 (in red), *Mferi* IVB14/OD_0535 (in blue), *Mferi* ΔMIB-MIP (in green), and *Mmc* GM12 YCp1.1 (in black). pH of the cultures of the wild-type strains at certain time-points is indicated. **C** Doubling time calculated during exponential phase of the four strains growing in standard SP5 medium at 37 °C without antibiotic selection. Results are the average of at least 3 biological replicates. *** *p* *<* 0.001, **** *p* < 0.0001.

To examine the activity of the MIB-MIP systems of *Mferi* we decided to generate a knock-out mutant strain. *Mferi* strain IVB14/OD_0535 has several genes coding for a total of four MIB-MIP tandem systems clustered in a single chromosomal locus^15^, in a similar disposition as in *Mmc* GM12^5^ (Fig. 4A). To assess activity of each unique MIB-MIP gene pair of *Mferi* we generated a MIB-MIP knock-out mutant (ΔMIB-MIP) by cloning and modifying the genome of *Mferi* in yeast prior transplantation of the modified genome to a new mycoplasma cell. The genome of *Mferi* IVB14/OD_0535 was transformed into *S. cerevisiae* carrying the necessary plasmids to replace the chromosomal locus coding for the four MIB-MIP gene pairs (MF5583_00301 to MF5583_00308, ~20 Kb) by a recombination template using the CReasPy-Cloning method^47^. The recombination template was designed to replace the sequence encompassed from the starting codon of the first MIB copy until the stop codon of the last MIP copy, preserving any upstream or downstream non-coding regions. YACs containing the modified genome were transplanted into an *Mcap* ΔRE recipient cell to obtain the desired *Mferi* ΔMIB-MIP strain (Supplementary Fig. S3). This knock-out mutant could grow with a certain delay compared to the WT strain in the absence of antibiotics (Fig. 4B), with a doubling time of 50 ± 2 min compared to 45 ± 3 min of the WT in SP5 (Fig. 4C). This growth delay is similar to the one observed when the *Mmc* GM12 strain is compared to the same strain carrying the YCp1.1 element, suggesting that insertion of this genetic region might be impacting bacterial fitness, not the absence of MIB-MIP genes.

### Development of replicative plasmids for *M. feriruminatoris*

**Fig. 5:**
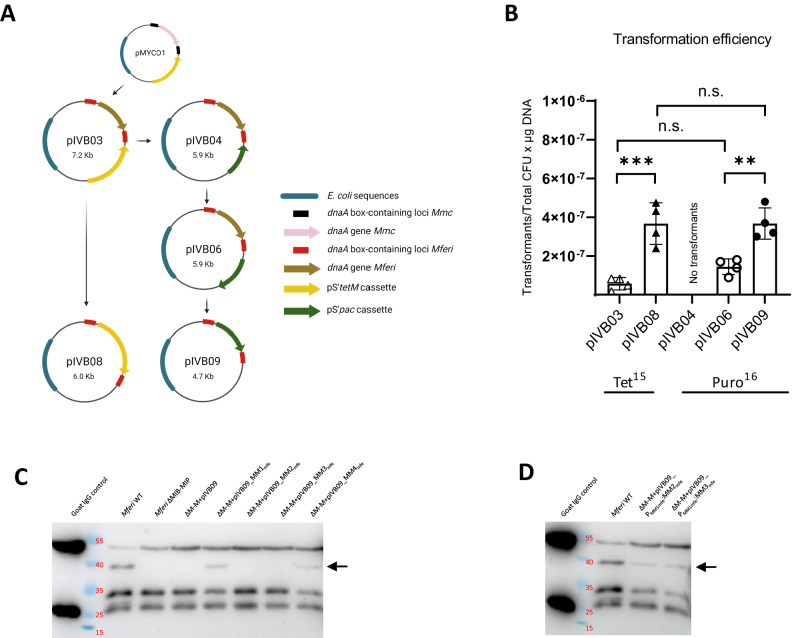
Construction of replicative *oriC*-plasmids for *M. feriruminatoris.* **A** Overview of the *oriC*- plasmids designed in this work. **B** Transformation efficiency upon introduction of the different shuttle vectors in *M. feriruminatoris*. A total of four independent biological replicates were conducted. Transformants with pIVB03 and pIVB08 were selected with 15 µg mL^−1^ tetracycline, while 16 µg mL^−1^ puromycin was used to select transformants with pIVB04, pIVB06 and pIVB09. Data was analyzed using one-way ANOVA tests with Tukey’s multiple comparisons test. ** *p* < 0.005, *** *p* *<* 0.001, n.s. non-significant. **C** IgG cleavage activity of several strains of *Mferi* determined by Western blot. Cleaved IgG heavy chain is indicated with an arrow. Strain *Mferi* ΔMIB-MIP is abbreviated as ΔM-M in this panel and in (**D**). **D** IgG cleavage activity in a MIB-MIP mutant strain can be restored with MIB-MIP pairs 2 and 3 if they are under the control of the P_MMmfe1_ promoter, measured by Western blot.

The genetic tools currently available for manipulation of *Mferi* are limited and only the introduction and modification of the bacterial genome in yeast has been reported, which is technically challenging and time consuming. To accelerate the shuttle in of genes into *Mferi*, we decided to generate replicative plasmids based on the origin of replication (*oriC*) sequence of the chromosome (Fig. 5A), as previously done for other *Mollicutes* species^51,58–60^. We adapted the *oriC*-plasmid pMYCO1 for use in *Mferi* by exchanging the *oriC* region of *Mmc* by the *oriC* region of *Mferi* strain G5847. This first *oriC*-plasmid, named pIVB03, could be successfully transformed in *Mferi* and promoted resistance to tetracycline at 15 µg mL^−1^. However, as *Mferi* strains engineered in yeast already have a tetracycline resistance cassette, we developed a derivative plasmid of pIVB03 named pIVB04 carrying the puromycin acetyl transferase (*pac*) gene, which was reported to confer resistance to puromycin in other closely related *Mollicutes* species^61^. This puromycin resistance-conferring plasmid was transformed but did not produce puromycin-resistant colonies carrying pIVB04 (Fig. 5B). This result confirmed that the plasmid backbone could indeed replicate in *Mferi*, but that the resistance cassette in pIVB04 was probably not expressed correctly. This negative outcome was changed by switching the direction of the *pac* gene (Fig. 5A), which became plasmid pIVB06, and allowed the recovery of resistant colonies at 16 µg mL^−1^ puromycin. In other *Mycoplasma* species, *oriC*-plasmids have been shown to be more stable when reducing the length of the *oriC* region or by deleting the *dnaA* gene^21,58^. Therefore, we modified the *oriC*-plasmids pIVB03 and pIVB06 by replacing the *dnaA* gene of *Mferi* by the antibiotic resistance cassette, generating streamlined *oriC*-plasmids with resistance to tetracycline (pIVB08) or to puromycin (pIVB09) (Fig. 5A). Note that we also switched the orientation of the *tetM* cassette in plasmid pIVB08. These plasmid versions transformed with an efficiency up to four times higher than their parental plasmids with *dnaA* counterparts (Fig. 5B). As *oriC*-plasmids from *Mollicutes* are known to be rapididly lost under non-selective conditions, we tested the stability of the pIVB09 vector in the population of *Mferi* upon serial passaging. The presence of the plasmid in the cells is heavily reduced in the absence of puromycin in the medium, but a small subpopulation of cells can retain it nevertheless (Supplementary Fig. S4). Thus, the constructed *oriC*-plasmids can be used for subsequent mutant phenotype complementation studies and as versatile backbones for heterologous expression systems reported in this study.

### Expression of native MIB-MIP gene pairs of *M. feriruminatoris* in trans

Each unique MIB-MIP gene pair of *Mferi* was cloned in a pIVB09 backbone under the control of their own natural promoters previously determined by 5’RACE. The newly constructed *Mferi* ΔMIB-MIP strain was transformed with each of these plasmids individually. Positive clones were exposed to goat IgGs to assess cleavage activity (Fig. 5C). Despite analyzing multiple clones harboring each MIB-MIP gene pair,we could only detect IgG heavy chain cleavage in clones expressing the first and last gene tandems of the cluster (MM1_mfe_ and MM4_mfe_), while other clones expressing the other MIB-MIP pairs (MM2_mfe_ and MM3_mfe_) showed marginal cleavage or activity below the detection limit. The activity of the promoters driving the expression of MM2_mfe_ and MM3_mfe_ could be confirmed by using plasmids carrying transcriptional fusions to a fluorescent reporter (Supplementary Fig. S5). As the transcriptomics data suggested that the MM1_mfe_ and MM4_mfe_ gene tandems were expressed at higher levels than MM2_mfe_ and MM3_mfe_, and that expression from the promoter region of MM1_mfe_ (P_MM1mfe_) had no apparent interplay with any rho-independent terminator or upstream regulatory sequences, we decided to reintroduce the MM2_mfe_ and MM3_mfe_ gene pairs under the control of P_MM1mfe_ in a ΔMIB-MIP genetic background. Under these conditions, all MIB-MIP gene pairs could be expressed, and mutants showed clear IgG cleavage activity (Fig. 5D), suggesting that all MIB-MIP gene pairs are functional *in cellulo*, and that the P_MM1mfe_ and the P_MM4mfe_ regions were sufficient to drive expression of two relatively large membrane-associated proteins organized as an operon in trans.

### *In cellulo* IgG cleavage by *M. hyopneumoniae* and *M. hyorhinis*

**Fig. 6:**
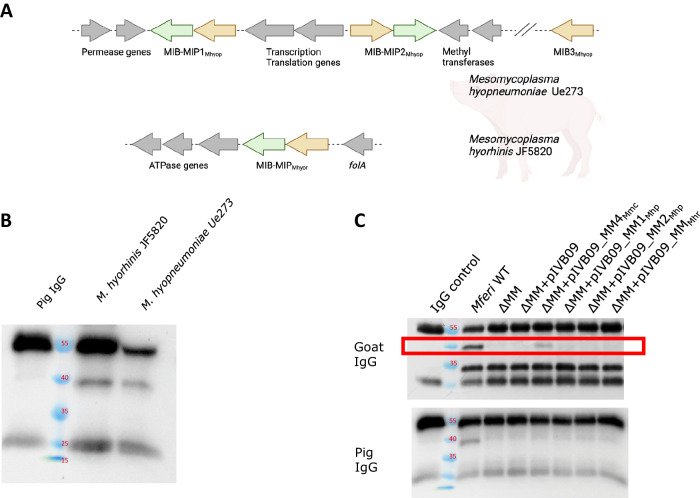
IgG cleavage activity in porcine *Mesomycoplasma* species. **A** Schematic representation of the chromosomal location of the MIB-MIP systems of *M. hyopneumoniae* and *M. hyorhinis*. **B** Porcine IgG cleavage activity of *M. hyopneumoniae* and *M. hyorhinis* analyzed by Western blot. **C** Porcine and goat IgG cleavage activity of *Mferi* ΔMIB-MIP strain expressing heterologous MIB-MIP systems.

Cleavage of immunoglobulins by the MIB-MIP system has been reported in *Mollicutes* of the formerly known “*Spiroplasma* phylogenetic group”, i.e., *Mmc*^10^ or *Mferi*^13^, but never in *Mollicutes* species like *Mesomycoplasma* spp. To determine if important porcine pathogens such as *M. hyopneumoniae* or *M. hyorhinis* can target and cleave host IgGs, we analyzed immunoglobulin cleavage activity of two strains isolated in Switzerland. Genome analysis revealed that *M. hyopneumoniae* Ue273 contains two complete MIB-MIP gene pairs and a single orphan MIB gene, while *M. hyorhinis* JF5820 only contains a single MIB-MIP gene pair^19^ (Fig. 6A). This contrasts with many *Mollicutes* species of the “*Spiroplasma* phylogenetic group”, where all MIB-MIP gene copies are clustered in a single chromosomal locus containing 3–4 complete MIB-MIP gene pairs. Interestingly, no ATPase gene cluster was found downstream of any of the different MIB-MIP copies in *M. hyopneumoniae*, as it is the case in most *Mollicutes* species. This ATPase gene cluster in *M. hyopneumoniae* is found in a different chromosomal location instead (Supplementary Fig. S6). Incubation with purified commercial IgGs isolated from naïve pig serum showed cleavage activity by both pathogens (Fig. 6B), with the heavy chain of the immunoglobulins being targeted.

### Heterologous expression of MIB-MIP gene pairs of other *Mollicutes*

The availability of a ΔMIB-MIP strain together with a vector capable of expression of MIB-MIP pairs in trans in *Mferi* prompted us to complement this strain with MIB-MIP systems from other *Mollicutes* species. First, we cloned the last gene tandem of the MIB-MIP operon of *Mmc* GM12 (MM4_Mmc_) under the control of the promoter region of the first gene tandem of the same species (P_MM1Mmc_), mimicking a similar disposition performed in situ at the chromosomal location in a previous work^10^. This construction was transformed in the ΔMIB-MIP strain and positive clones exhibit restored capacity to cleave goat IgGs (Fig. 6C, lane ΔM/M+pIVB09-MM4_Mmc_). Hereafter, we cloned the two complete MIB-MIP gene pairs and the single MIB-MIP gene tandem from *M. hyopneumoniae* Ue273 (MM1_Mhp_ and MM2_Mhp_) and *M. hyorhinis* JF5820 (MM_Mhr_) in a pIVB09 backbone. In a first attempt, we introduced each MIB-MIP set under the control of their natural promoters, as previously done with the MIB-MIP genes of *Mferi* and *Mmc*. However, despite obtaining similar number of transformants carrying the different *oriC*-plasmids, no IgG cleavage was detected (Fig. 6C). To facilitate recombinant expression, we adapted all the MIB-MIP coding sequences to the codon usage of *Mmc*, the closest species of the “*Mycoplasma mycoides* cluster” to *Mferi* with an available characterized codon usage table (kazusa.or.jp) and replaced the natural promoters with the P_MM1mfe_, which proved capable of generating mRNA of similar length as previously shown in this study. However, transformants carrying these new constructs could neither cleave goat nor porcine IgGs (Fig. 6C), suggesting that the system was not active or could not be correctly exported, folded, or displayed at the membrane of *Mferi* cells. Interestingly, the wild-type strain of *Mferi* could bind and cleave porcine IgGs, despite the pig not being its natural animal host.

**Fig. 7:**
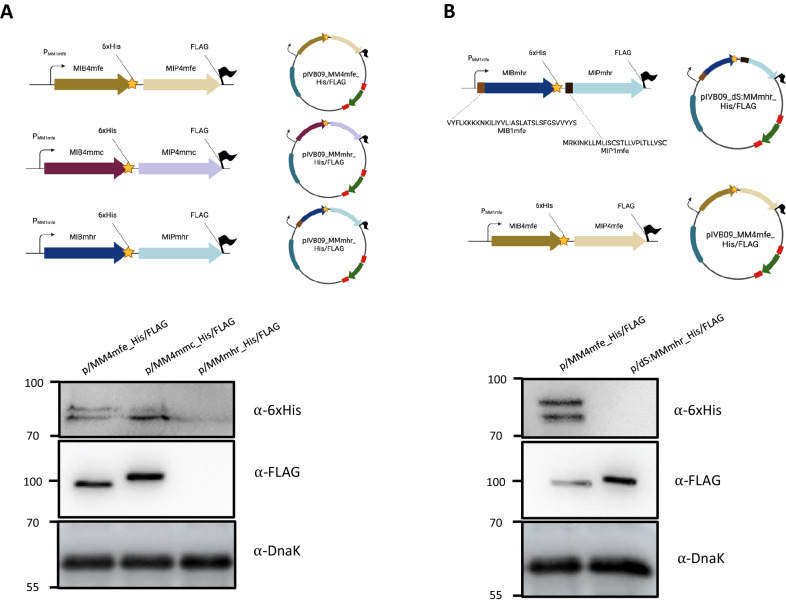
Heterologous MIB-MIP expression analysis in *Mferi.* **A** Schematic representation of three plasmids carrying tagged versions of MIB-MIP copies from different species (top). Detection of 6xHis and FLAG-tagged MIB and MIP, respectively. DnaK was used as a loading control (bottom). **B** Schematic representation of the two plasmids used to assess the role of the signal peptides in the expression of the MIB-MIP system of *M. hyorhinis* (top). Detection of 6xHis and FLAG-tagged MIB and MIP, respectively. DnaK was used as a loading control (bottom).

To further investigate this, we cloned in pIVB09 tagged-versions of the MIB-MIP systems of *Mferi* (MM4_Mfe_), *Mmc* (MM4_Mmc_), and the single MIB-MIP system of *M. hyorhinis* (MM_Mhr_) to track protein expression by immunoblotting. All MIB genes were fused with a C-terminal 6xHis tag, while their MIP counterparts were tagged with a C-terminal FLAG tag. Analysis of *Mferi* ΔMIB-MIP strains carrying these plasmids showed that neither of the proteins forming the MIB-MIB system of *M. hyrorhinis* was expressed in these conditions (Fig. 7A), which explained the lack of IgG cleavage showed previously. Sequence analysis of the MIB-MIP systems of *Mferi*, *Mmc,* and *M. hyorhinis* showed significant differences in the N-terminal residues (Supplementary Fig. S7), which could prevent export of these proteins to the cell surface. To test this, we replaced the predicted signal peptides of the tagged MIB-MIP system of *M. hyorhinis* with the ones present in the MIB-MIP pair 1 of *Mferi*. Strains carrying this construction could correctly express the protease component of the MM_Mhr_, but not the binding protein (Fig. 7B). We also corroborated that gene complementation with a single functional partner (either MIB or MIP) cannot restore IgG cleavage capacity in the ΔMIB-MIP mutant (Supplementary Fig. S8).

### Goats infected intranasally and transtracheally with *Mycoplasma feriruminatoris* did not develop disease

**Fig. 8:**
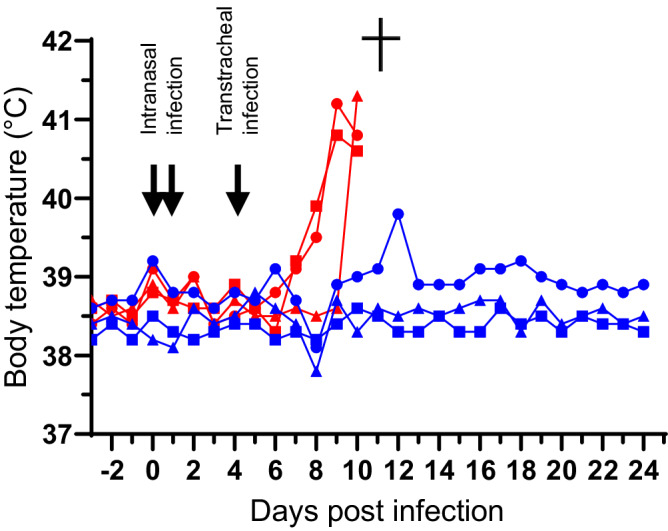
Monitoring of body temperature during the animal challenge. Two groups of three outbreed goats were infected with either *Mccp* ILRI181 (in red) or *Mferi* G5847^T^ (in blue). Body temperature was monitored daily for both groups until experiment termination. Goats infected with *Mccp* were euthanized at day 10 due to the severity of their clinical signs, as indicated with a cross.

*Mferi* is considered a promising candidate for the development of a vaccine chassis^13^. However, *Mferi* has only been isolated from wild *caprinae*^11^; thus, data regarding its pathogenic potential in closely related domestic animals are absent. Therefore, we decided to assess the pathogenicity of the type-strain G5847^T^ of *Mferi* as the representative member of the species. We used a challenge model established for the phylogenetically related species *Mmc*^9^ and modified for *Mccp*^55^, which is robust and reproducible^62^. Positive control was the highly virulent *Mccp* ILRI181^63^. Clinical evaluation was assessed daily and was carried out 10 days pre-infection up to 25 days post-infection (dpi). Goats infected with *Mferi* showed no clinical signs in contrast to animals infected with *Mccp*, which showed onset of clinical disease including elevated body temperature at 6–8 dpi (Fig. 8). This was followed by high fever (>40.5 °C for all animals), associated with respiratory distress, coughing, and wheezing (8–10 dpi), less movement and reduced intake of food. All criteria considered, this clinical evaluation led to a severity grade of 3 at 10 dpi; consequently, the three animals infected with *Mccp* were euthanized (Supplementary Fig. S8). All animals infected with either species did not show a clear difference in the hematological parameters compared to their baseline levels prior infection (Supplementary Fig. S9). Postmortem analysis did reveal contagious caprine pleuropneumonia (CCPP) typical pathomorphological changes including the detection of *Mccp*, while the animals infected with *Mferi* did not have any lesions pointing towards *Mferi*-related disease and *Mferi* could not be isolated from the animals.

## Discussion

*Mferi* has so far only been isolated from wild ruminants such as Alpine ibex^11,12^. The ability to modify its genome using synthetic genomics tools^13^, the absence of the cell wall, its expected glycosylation capacity (as observed in other *Mollicutes* species^64^), and its favorable growth attributes make it an appealing candidate for vaccine- and drug delivery especially in reference to the respiratory tract or cancer treatment^65^.

Surface expression of heterologous antigens in *Mferi* is desirable for future antigen presentation in a live vaccine chassis. We aimed to investigate heterologous surface expression and focused our work on the MIB-MIP system. To get an idea of the sequence conservation of the MIB-MIP system in our model strain, we analyzed the presence and genetic identity of the MIB-MIP system in our strain and compared it to other different species. It has been shown that several copies of such genes, homologous to the MIB-MIP system of *Mmc*, were frequently present in most species of *Mycoplasma*, *Mycoplasmopsis*, *Metamycoplasma* and *Mesomycoplasma*, and a token presence in *Mycoplasmoides* spp.^5^. Our *Mferi* strain IVB14/OD_0535 contains 4 copies of MIB-MIP pairs all clustered in the same genomic location, in a similar disposition as in *Mmc* GM12. Moreover, they are phylogenetically very closely related, in contrast with the MIB-MIP system present in *Mferi* type strain G5847^T^. This fact suggests that these genes may have distinct origin between different isolates.

Our transcriptomics and 5’RACE analyses show that each MIB-MIP pair may function as an individual transcriptional unit—an operon—with its own putative promoter and a short palindromic sequence resembling rho-independent terminator sequences. We showed that the promoter of the first and last gene tandems are significantly stronger than the other two, which may influence the transcription of downstream elements. The first promoter element may overcome the terminator element and drive the expression of other MIB-MIP pairs, as shown by the 5’RACE result of MIB2 (Fig. 3 and Supplementary Fig. 2). Despite the data presented here, it cannot be ruled out that other internal promoter regions exist and are used to transcribe the MIP-encoding genes, especially in the case of MIP3. As there is no putative rho-independent terminator after the coding region of MIP4, the last promoter likely plays a role on the expression of the highly conserved putative ATP-synthase gene cluster situated immediately downstream. It is still not clear if MIB-MIP-related IgG cleavage requires the activity of the downstream ATPase, but our results show that the two gene clusters do not necessarily work *in cis*. In line with this, the MIB-MIP systems present in *M. hyopneumoniae* are unlinked to the ATPase gene cluster (Fig. 6A and Supplementary Fig. S6), which is still present but in a different genetic context in this species, and it has its own putative promoter. The putative promoter region of the ATPase gene cluster of *M. hyopneumoniae* might consist of a classic −10 region and relatively canonical −35 box, separated by a large series of Adenines. This configuration resembles others found in promoters with DNA slippage control in several *Mollicutes*^66^. Thus, it is debatable that independent promoter regions can drive the expression of these ATPase related genes, even in *Mollicutes* species where this cluster is located downstream of the MIB-MIP genes. Due to their reduced-size genomes, *Mollicutes* are thought to be devoid of many transcriptional regulatory elements, and rho-independent terminators have been suggested as major fine-tuning, transcription-controlling elements^67^, in conjunction with DNA supercoiling and RNA degradation^68^.

Here, we developed transformation protocols and *oriC*-type plasmid vectors to shuttle antigen-encoding genes into *Mferi* and to accelerate the testing of heterologous protein expression. Our growth curves of the wild-type strain of *Mferi* showed the rapid decline of viable cells after 20 h of cultivation in SP5 medium, coinciding with medium acidification below pH 7. This characteristic contrasts slightly with *Mmc*, a closely related, relatively fast-growing *Mycoplasma* species, which can survive and maintain high bacterial titers for longer time in acidic conditions. Loss of viability upon acidification in mycoplasmas is not exceptional^69^, thus the differences in low pH tolerance between *Mferi* and *Mmc* could be attributed to distinctive metabolic capabilities^11^ or growth requirements in both species. This reduced tolerance towards lower pH prompted us to adapt the standard transformation protocols for mycoplasmas of the “*M. mycoides* cluster”, which use cells harvested at pH 6.2–6.5 and PEG solutions buffered at a similar pH, for transformation of *Mferi*. Most transformable *Mollicutes* species have higher transformation efficiencies when harvested at late-log phase^70^. By increasing the initial pH of the SP5 medium from 7.5 to 8, we could significantly increase the bacterial titers of *Mferi* after an overnight growth to 1–3 × 10^9^ CFU mL^−1^ at pH 7, right before cell titer decline, optimizing transformation efficiencies for this bacterium. Thus, we also adapted the pH of all transformation solutions to pH 7 to mimic the medium conditions at the harvesting point. When comparing growth of the *Mferi* WT strain with the MIB-MIP mutant strain we observed a small delay. However, this delay is also observed between the *Mmc* GM12 strain and the *Mmc* YCp1.1, in which no gene has been knocked out. This fact suggests that the presence of the YCp1.1 element in some mycoplasma genomes can have an impact on growth. It has been previously shown in other species that the presence of certain antibiotic markers influences mycoplasma growth even in the absence of the drug^71^, which might mask some mutant phenotypes. Thus, removal of antibiotic cassettes is highly advisable in future functional genomics studies in *Mferi*.

In this work, we also developed a series of replicative plasmids based on the modification of the origin of replication of the type-strain G5847^T^ that can be easily used in *Mferi*. Many species of *Mollicutes* can stably maintain episomal DNA containing the *oriC* sequence of the same species or a closely related one^21,51,58–60,72–74^. For certain species such as *M. agalactiae*, it has been found that the *dnaA* gene present in these *oriC* plasmids is not essential for replication and propagation. The removal of *dnaA* and simplification of the *oriC* region results in less frequent integration events at the chromosomal *oriC* locus in most species^21,58^, with the cost of usually lower transformation efficiency rates. On the contrary, in the case of *oriC*-plasmids derived from *Mferi*, removal of the *dnaA* gene resulted in 5 times higher transformation efficiencies regardless of the antibiotic marker used. Moreover, plasmids are rapidly lost in the absence of antibiotic pressure within the first passages (Supplementary Fig. S4), although it can stably remain in a small number of cells, suggesting spontaneous chromosomal integration. Only one *oriC*-plasmid developed in this work, pIVB04, in which the *tetM* marker had been replaced with *pac* marker did not yield any transformants despite having the same configuration as pIVB03 (Fig. 5). It was only when the orientation of the *pac* marker was flipped (plasmid pIVB06) that the plasmid yielded a similar number of transformants than pIVB03. This fact suggests that the *oriC* region contains promoter sequences that could challenge the transcription of the pS’*pac* cassette by antisense inhibition of gene expression. Antisense RNA-mediated transcriptional attenuation in bacteria is well described^75–77^, involving either dsRNA-specific RNases, peptide nucleic acids, phosphorodiamidate morpholino oligomers or just by steric hindrance of transcription or translation. In *Mollicutes*, antisense RNAs have been identified in pathogenic species of swine such as *M. hyopneumoniae*^78^ and human species like *M. pneumoniae*^79^ or *M. genitalium*^80^, and their role in modulation of gene expression has been acknowledged. However, it seems likely that by steric obstruction, the RNA polymerase complex cannot successfully read through two genes with colliding orientations if both promoters are spatially close, which should result in a lower expression of both transcripts. This was shown in *M. genitalium*, when transposons expressing the toxic MG_428 gene coding for the alternative sigma factor σ^20^ were all inserted in highly expressed genes in the opposite orientation of transcription, which dampened expression of the toxic gene and allowed cells to live^81,82^. In the case of pIVB04, most likely the promoter of the *dnaN* gene is interfering with the expression of the *pac* marker controlled by the spiralin promoter pS’, interfering with the expression of the antibiotic cassette and limiting the puromycin resistance of transformed cells with the *oriC*-plasmid. This is not the case for pIVB03 and pMYCO1 *oriC*-plasmids, likely due to the larger size of the *tetM* marker (1.9 kb) compared to the *pac* cassette (0.6 kb). By using these *oriC*-plasmids, we could demonstrate that the promoter elements of the first MIB-MIP gene copy of *Mferi* (P_MM1mfe_) was capable of successfully drive the expression of all MIB-MIP gene tandems individually in trans, which makes it a promising tool for recombinant expression of other rather large membrane proteins using this bacterium in the future.

Despite several attempts, expression of active heterologous MIB-MIP systems could only be achieved with a MIB-MIP system of *Mmc*, which is phylogenetically closely related to *Mferi*. Expression of functional MIB-MIP gene tandems from the porcine *Mollicutes* species *M. hyopneumoniae* and *M. hyorhinis* was not possible, despite the use of native promoters from *Mferi* or adapting the codon usage. The structure of the MIB-MIP tandem system has been recently obtained and characterized^10^ and shows direct contacts between the two protein counterparts and with the targeted immunoglobulin. Therefore, correct export and folding of both proteins is likely pivotal for the system to work. We show that IgG cleavage of *M. hyopneumoniae* and *M. hyorhinis in cellulo* is possible under the standard laboratory conditions, which indicates that the systems present in these bacteria are active when expressed correctly. Despite our efforts, we failed at expressing active MIB-MIP systems from distantly related *Mollicutes* in *Mferi*, most likely due to problems related to the export of the system to the membrane. Despite many years of research, the protein export systems of *Mollicutes* are poorly characterized^83^. Most MIP proteins analyzed in this work contain a classic lipoprotein signal peptide (type II signal peptide), which consist of a positively charged N-terminal region, followed by a central hydrophobic area and a polar C-terminal region (lipobox)^84^. This signal peptide is also known to contain a preserved motif LXXC, which is recognized by the preprolipoprotein diacylglyceryl transferase (Lgt, encoded in MF5583_00077 and 00079 in *Mferi* IVB14/OD_0535) followed by the apolipoprotein *N-*acyltransferase (Lnt, encoded in MF5583_00341) that will create the linkage of the protein to the cell membrane after translocation via the Sec pathway^85^. However, none of the MIB proteins analyzed have a similar type II signal peptide or any clear transmembrane domain that suggests in silico association at the cell surface (Supplementary Fig. S10), aside from the interaction with MIP required for immunoglobulin cleavage. Furthermore, very low MIB protein levels are detected in our proteomics analyses in either *Mmc* or *Mferi*, as it was previously reported in *Mmc*^5^. This might be due to technical problems like low solubilization of MIB proteins during sample preparation or lack of suitable resulting peptides for MS analysis. However, in another closely related *Mycoplasma* species namely *Mmm*, also only the MIP proteins have been clearly detected in the surface proteome, while MIB proteins can only be seldomly detected^86^. Similarly, the closely related protein M, present in other *Mollicutes* species usually devoid of MIB-MIP systems^5^, also lacks any clear membrane anchoring signal and could not be identified in the protein membrane enriched fractions or cell surface protein labeling in a thorough proteomics study carried out in *M. genitalium*^87^. However, a recent study characterizing the protein M homolog (IbpM) from *M. pneumoniae* shows data indicating that this protein is located at the cell surface^88^, despite that advanced transmembrane domain predictors like DeepTMHMM^89^ do not predict the presence of any transmembrane domain in neither protein M nor MIB. However, experimental data indicates that both MIB and MIP are associated to the membrane in *Mferi* (Supplementary Fig. S10) and also in *M. bovis*^8^. Understanding how these and other proteins lacking conventional signal peptides are exported in *Mferi* is crucial to develop functional display systems in this bacterium.

Finally, we investigated the pathogenicity of *Mferi* in domestic goats using an infection model that has been successfully used for phylogenetically closely related mycoplasmas. *Mollicutes* are reported to have high species tropism this should be confirmed for *Mferi* in an in vivo experiment. Our data do not point to pathogenicity in domestic goats and therefore this organism is unlikely to infect even phylogenetically more distant organisms, which is important for safety concerns. Only one infection route was tested, which is the main one in closely related bacteria of the “*M. mycoides* cluster”^90^. Goats challenged with *Mccp*, as expected, reached endpoint criteria at 10 dpi and were euthanized. *Mferi* could not be isolated from animals challenged with the latter, while *Mccp* was isolated from pathomorphological lesion typical of CCPP of the animals infected with it. The absence of *Mferi* from the post-mortem tissues investigated supports the fact that the animals cleared *Mferi* from the system.

In conclusion, we assessed pathogenicity of *Mferi* in an established animal model, developed new *oriC*-based vectors for rapid and versatile gene delivery in this microorganism, and use them to characterize expression of native and foreign anti-immunoglobulin systems of *Mollicutes*. This study provides new data regarding the molecular mechanisms of these specialized machineries that should aid in the understanding of the immune evasion strategies of pathogenic *Mollicutes* species. Moreover, we identified promoters suitable to drive expression of large heterologous surface proteins, which will be pivotal for future applications of *Mferi* as a bacterial vaccine chassis.

### Supplementary information

Supplementary Material

Description of Additional Supplementary Files

Supplementary Data 1

Supplementary Data 2

Supplementary Data 3

Reporting summary

## Data Availability

All sequencing related data can be found in BioProject PRJNA1062711 or upon request. Mass spectrometry-based proteomics data can be found in Supplementary Data 2 and via ProteomeXchange with identifier PXD053286. Uncropped blots are shown in the Supplementary Fig. S11. All plasmid sequences can be found in Supplementary Data 3. All raw data used to generate the graphs displayed in this manuscript as well as the raw RNAseq data can be obtained as downloadable files from Figshare^91^.

## References

[1] Namba, S. Molecular and biological properties of phytoplasmas. *Proc. Jpn. Acad. Ser. B. Phys. Biol. Sci*. **95**, 401–418 (2019). 10.2183/pjab.95.028 PMC6766451 31406061

[2] Fischer A (2012). The origin of the ‘*Mycoplasma mycoides* cluster’ coincides with domestication of ruminants. PLoS One.

[3] Taylor-Robinson D, Jensen JS (2011). *Mycoplasma genitalium*: from chrysalis to multicolored butterfly. Clin. Microbiol. Rev..

[4] Waites KB, Talkington DF (2004). *Mycoplasma pneumoniae* and its role as a human pathogen. Clin. Microbiol. Rev.

[5] Arfi Y (2016). MIB-MIP is a mycoplasma system that captures and cleaves immunoglobulin G. Proc. Natl Acad. Sci. USA.

[6] Guiraud J (2023). Improved transformation efficiency in *Mycoplasma hominis* enables disruption of the MIB–MIP system targeting human immunoglobulins. Microbiol. Spectr..

[7] Arfi Y, Lartigue C, Sirand-Pugnet P, Blanchard A (2021). Beware of mycoplasma anti-immunoglobulin strategies. mBio.

[8] Zhao H (2021). MBOVPG45_0375 encodes an IgG-binding protein and MBOVPG45_0376 encodes an IgG-cleaving protein in *Mycoplasma*
*bovis*. Front. Vet. Sci..

[9] Jores J (2019). Removal of a subset of non-essential genes fully attenuates a highly virulent *Mycoplasma* strain. Front. Microbiol..

[10] Nottelet P (2021). The mycoplasma surface proteins MIB and MIP promote the dissociation of the antibody-antigen interaction. Sci. Adv..

[11] Jores J (2013). *Mycoplasma feriruminatoris* sp. nov., a fast growing *Mycoplasma* species isolated from wild Caprinae. Syst. Appl. Microbiol..

[12] Ambroset C, Pau-Roblot C, Game Y, Gaurivaud P, Tardy F (2017). Identification and characterization of *Mycoplasma feriruminatoris* sp. nov. strains isolated from Alpine ibex: A 4th species in the *Mycoplasma mycoides* cluster hosted by non-domesticated ruminants?. Front. Microbiol..

[13] Talenton V (2022). Genome engineering of the fast-growing *Mycoplasma feriruminatoris* toward a live vaccine chassis. ACS Synth. Biol..

[14] Labroussaa, F., Torres-Puig, S. & Jores, J. Chapter 1 - Genome transplantation in *Mollicutes*. In *Methods in Microbiology*, Vol. 52 (eds Gurtler, V. & Calcutt, M.) 3–32 (Academic Press, 2023).

[15] Labroussaa F, Thomann A, Nicholson P, Falquet L, Jores J (2020). Complete genome sequence of *Mycoplasma feriruminatoris* Strain IVB14/OD_0535, isolated from an Alpine Ibex in a Swiss Zoo. Microbiol. Resour. Announc..

[16] Garrido V (2021). Engineering a genome-reduced bacterium to eliminate *Staphylococcus aureus* biofilms in vivo. Mol. Syst. Biol..

[17] Mazzolini R (2023). Engineered live bacteria suppress *Pseudomonas aeruginosa* infection in mouse lung and dissolve endotracheal-tube biofilms. Nat. Biotechnol..

[18] Sambrook, J., Fritsch, E. F. & Maniatis, T. *Molecular Cloning: A Laboratory Manual* (Cold Spring Harbor Laboratory Press, 1989).

[19] Trueeb BS, Gerber S, Maes D, Gharib WH, Kuhnert P (2019). Tn-sequencing of *Mycoplasma hyopneumoniae* and *Mycoplasma hyorhinis* mutant libraries reveals non-essential genes of porcine mycoplasmas differing in pathogenicity. Vet. Res..

[20] Labroussaa F (2016). Impact of donor-recipient phylogenetic distance on bacterial genome transplantation. Nucleic Acids Res..

[21] Cordova CMM (2002). Identification of the origin of replication of the *Mycoplasma pulmonis* chromosome and its use in *oriC* replicative plasmids. J. Bacteriol..

[22] Friis NF (1975). Some recommendations concerning primary isolation of *Mycoplasma suipneumoniae* and *Mycoplasma flocculare* a survey. Nord. Vet. Med..

[23] Lartigue C (2009). Creating bacterial strains from genomes that have been cloned and engineered in yeast. Science.

[24] Katoh K, Standley DM (2013). MAFFT multiple sequence alignment software version 7: improvements in performance and usability. Mol. Biol. Evol..

[25] Nguyen L-T, Schmidt HA, von Haeseler A, Minh BQ (2015). IQ-TREE: a fast and effective stochastic algorithm for estimating maximum-likelihood phylogenies. Mol. Biol. Evol..

[26] Minh BQ (2020). IQ-TREE 2: new models and efficient methods for phylogenetic inference in the genomic era. Mol. Biol. Evol..

[27] Hoang DT, Chernomor O, Von Haeseler A, Minh BQ, Vinh LS (2018). UFBoot2: improving the ultrafast bootstrap approximation. Mol. Biol. Evol..

[28] Kalyaanamoorthy S, Minh BQ, Wong TKF, Von Haeseler A, Jermiin LS (2017). ModelFinder: fast model selection for accurate phylogenetic estimates. Nat. Methods.

[29] Hill V (2021). Minimalistic mycoplasmas harbor different functional toxin-antitoxin systems. PLoS Genet..

[30] Wick RR, Judd LM, Gorrie CL, Holt KE (2017). Unicycler: resolving bacterial genome assemblies from short and long sequencing reads. PLoS Comput. Biol..

[31] Seemann T (2014). Prokka: rapid prokaryotic genome annotation. Bioinformatics.

[32] Danecek P (2021). Twelve years of SAMtools and BCFtools. Gigascience.

[33] Wang L, Wang S, Li W (2012). RSeQC: quality control of RNA-seq experiments. Bioinformatics.

[34] Braga-Lagache S (2016). Robust label-free, quantitative profiling of circulating plasma microparticle (MP) associated proteins. Mol. Cell. Proteom..

[35] Buchs N (2018). Absolute quantification of grapevine red blotch virus in grapevine leaf and petiole tissues by proteomics. Front Plant Sci..

[36] Deutsch EW (2010). A guided tour of the trans-proteomic pipeline. Proteomics.

[37] Eng JK (2015). A deeper look into Comet—implementation and features. J. Am. Soc. Mass Spectrom..

[38] Craig R, Beavis RC (2003). A method for reducing the time required to match protein sequences with tandem mass spectra. Rapid Commun. Mass Spectrom..

[39] Kong AT, Leprevost FV, Avtonomov DM, Mellacheruvu D, Nesvizhskii AI (2017). MSFragger: ultrafast and comprehensive peptide identification in mass spectrometry–based proteomics. Nat. Methods.

[40] Kim S, Pevzner PA (2014). MS-GF+ makes progress towards a universal database search tool for proteomics. Nat. Commun..

[41] Tabb DL, Fernando CG, Chambers MC (2007). MyriMatch: highly accurate tandem mass spectral peptide identification by multivariate hypergeometric analysis. J. Proteome Res..

[42] Choi H, Ghosh D, Nesvizhskii AI (2008). Statistical validation of peptide identifications in large-scale proteomics using the target-decoy database search strategy and flexible mixture modeling. J. Proteome Res..

[43] Shteynberg D (2011). iProphet: multi-level integrative analysis of shotgun proteomic data improves peptide and protein identification rates and error estimates. Mol. Cell Proteom..

[44] Zybailov BL, Florens L, Washburn MP (2007). Quantitative shotgun proteomics using a protease with broad specificity and normalized spectral abundance factors. Mol. Biosyst..

[45] Zhang Y, Wen Z, Washburn MP, Florens L (2010). Refinements to label free proteome quantitation: how to deal with peptides shared by multiple proteins. Anal. Chem..

[46] Perez-Riverol Y (2022). The PRIDE database resources in 2022: a hub for mass spectrometry-based proteomics evidences. Nucleic Acids Res..

[47] Ruiz E (2019). CReasPy-cloning: a method for simultaneous cloning and engineering of megabase-sized genomes in yeast using the CRISPR-Cas9 system. ACS Synth. Biol..

[48] Tsarmpopoulos I (2016). In-yeast engineering of a bacterial genome using CRISPR/Cas9. ACS Synth. Biol..

[49] Gietz RD, Schiestl RH, Willems AR, Woods RA (1995). Studies on the transformation of intact yeast cells by the LiAc/SS-DNA/PEG procedure. Yeast.

[50] Kouprina N, Larionov V (2008). Selective isolation of genomic loci from complex genomes by transformation-associated recombination cloning in the yeast *Saccharomyces cerevisiae*. Nat. Protoc..

[51] Lartigue C, Blanchard A, Renaudin J, Thiaucourt F, Sirand-Pugnet P (2003). Host specificity of mollicutes *oriC* plasmids: functional analysis of replication origin. Nucleic Acids Res..

[52] Puigbò P, Guzmán E, Romeu A, Garcia-Vallvé S (2007). OPTIMIZER: a web server for optimizing the codon usage of DNA sequences. Nucleic Acids Res..

[53] Bordier C (1981). Phase separation of integral membrane proteins in Triton X-114 solution. J. Biol. Chem..

[54] Schumacher M (2019). Evidence for the cytoplasmic localization of the L-α-glycerophosphate oxidase in members of the ‘*Mycoplasma mycoides* Cluster’. Front. Microbiol..

[55] Liljander A (2019). Reproduction of contagious caprine pleuropneumonia reveals the ability of convalescent sera to reduce hydrogen peroxide production in vitro. Vet. Res..

[56] Yus E (2012). Transcription start site associated RNAs in bacteria. Mol. Syst. Biol..

[57] Gruber AR, Lorenz R, Bernhart SH, Neuböck R, Hofacker IL (2008). The Vienna RNA websuite. Nucleic Acids Res..

[58] Sharma S (2015). Development and host compatibility of plasmids for two important ruminant pathogens, *Mycoplasma bovis* and *Mycoplasma agalactiae*. PLoS One.

[59] Maglennon GA (2013). Development of a self-replicating plasmid system for *Mycoplasma hyopneumoniae*. Vet. Res..

[60] Ye F, Renaudin J, Bové JM, Laigret F (1994). Cloning and sequencing of the replication origin (*oriC*) of the *Spiroplasma citri* chromosome and construction of autonomously replicating artificial plasmids. Curr. Microbiol.

[61] Algire MA (2009). New selectable marker for manipulating the simple genomes of *Mycoplasma* species. Antimicrob. Agents Chemother..

[62] Jores J (2020). Contagious bovine and caprine pleuropneumonia: a research community’s recommendations for the development of better vaccines. npj Vaccines.

[63] Falquet L (2014). Complete genome sequences of virulent *Mycoplasma capricolum* subsp. *capripneumoniae* Strains F38 and ILRI181. Genome Announc..

[64] Daubenspeck JM, Jordan DS, Simmons W, Renfrow MB, Dybvig K (2015). General N-and O-linked glycosylation of lipoproteins in mycoplasmas and role of exogenous oligosaccharide. PLoS One.

[65] Lim B (2022). Reprogramming synthetic cells for targeted cancer therapy. ACS Synth. Biol..

[66] Citti C, Nouvel L-X, Baranowski E (2010). Phase and antigenic variation in Mycoplasmas. Future Microbiol..

[67] Mazin PV (2014). Transcriptome analysis reveals novel regulatory mechanisms in a genome-reduced bacterium. Nucleic Acids Res..

[68] Yus E (2019). Determination of the gene regulatory network of a genome-reduced bacterium highlights alternative regulation independent of transcription factors. Cell Syst..

[69] Oral Mucosal Microbes. *Atlas of Oral Microbiology*. 95–107 10.1016/B978-0-12-802234-4.00005-7 (2015).

[70] Minion FC, Kapke PA (1998). Transformation of mycoplasmas. Methods Mol. Biol..

[71] Pich OQ, Burgos R, Planell R, Querol E, Piñol J (2006). Comparative analysis of antibiotic resistance gene markers in *Mycoplasma genitalium*: application to studies of the minimal gene complement. Microbiology.

[72] Shahid MA, Marenda MS, Markham PF, Noormohammadi AH (2014). Development of an *oriC* vector for use in *Mycoplasma synoviae*. J. Microbiol Methods.

[73] Janis C (2005). Versatile use of *oriC* plasmids for functional genomics of *Mycoplasma capricolum* subsp. *capricolum*. Appl. Environ. Microbiol.

[74] Lee S-W, Browning GF, Markham PF (2008). Development of a replicable oriC plasmid for *Mycoplasma gallisepticum* and *Mycoplasma imitans*, and gene disruption through homologous recombination in *M. gallisepticum*.. Microbiology.

[75] Rasmussen LCV, Sperling-Petersen HU, Mortensen KK (2007). Hitting bacteria at the heart of the central dogma: sequence-specific inhibition. Micro. Cell Fact..

[76] Nekhotiaeva N, Awasthi SK, Nielsen PE, Good L (2004). Inhibition of *Staphylococcus aureus* gene expression and growth using antisense peptide nucleic acids. Mol. Ther..

[77] Brantl S, Wagner EGH (2002). An antisense RNA-mediated transcriptional attenuation mechanism functions in *Escherichia coli*. J. Bacteriol..

[78] Siqueira FM (2016). Mycoplasma non-coding RNA: identification of small RNAs and targets. BMC Genom..

[79] Lloréns-Rico V (2016). Bacterial antisense RNAs are mainly the product of transcriptional noise. Sci. Adv..

[80] Lluch-Senar M, Vallmitjana M, Querol E, Piñol J (2007). A new promoterless reporter vector reveals antisense transcription in *Mycoplasma genitalium*. Microbiology.

[81] Torres-Puig S, Broto A, Querol E, Piñol J, Pich OQ (2015). A novel sigma factor reveals a unique regulon controlling cell-specific recombination in *Mycoplasma genitalium*. Nucleic Acids Res..

[82] Torres-Puig S (2018). Activation of σ20-dependent recombination and horizontal gene transfer in *Mycoplasma genitalium*. DNA Res..

[83] Gaurivaud P, Tardy F (2022). The Mycoplasma spp. ‘Releasome’: a new concept for a long-known phenomenon. Front. Microbiol..

[84] Kaushik S, He H, Dalbey RE (2022). Bacterial signal peptides-navigating the journey of proteins. Front. Physiol..

[85] Nakayama H, Kurokawa K, Lee BL (2012). Lipoproteins in bacteria: structures and biosynthetic pathways. FEBS J..

[86] Krasteva I (2014). Characterization of the in vitro core surface proteome of *Mycoplasma mycoides* subsp. *mycoides*, the causative agent of contagious bovine pleuropneumonia. Vet. Microbiol.

[87] Párraga-Niño N, Colomé-Calls N, Canals F, Querol E, Ferrer-Navarro M (2012). A comprehensive proteome of *Mycoplasma genitalium*. J. Proteome Res.

[88] Blötz C, Singh N, Dumke R, Stülke J (2020). Characterization of an immunoglobulin binding protein (IbpM) from Mycoplasma pneumoniae. Front. Microbiol..

[89] Hallgren, J. et al. DeepTMHMM predicts alpha and beta transmembrane proteins using deep neural networks. *bioRxiv*10.1101/2022.04.08.487609 (2022).

[90] Cottew GS (1987). Taxonomy of the *Mycoplasma mycoides* cluster. Isr. J. Med. Sci..

[91] Torres-Puig, S. et al. Functional surface expression of immunoglobulin cleavage systems in a candidate Mycoplasma vaccine chassis—Figure Data. *FigShare*10.6084/m9.figshare.25902661 (2024). 10.1038/s42003-024-06497-8 PMC11213901 38942984

