# Supplementary material for: Functional surface expression of immunoglobulin cleavage systems in a candidate Mycoplasma vaccine chassis
Source: Commun Biol. 2024 Jun 28;7:779. doi: 10.1038/s42003-024-06497-8 (PMC11213901; doi:10.1038/s42003-024-06497-8)
Supplement: Supplementary file 1 — Supplementary Material [file 42003_2024_6497_MOESM1_ESM.pdf]

Table S1. List of primers used in this work

Primer code	Primer name	Primer sequence	Purpose
001	oriG5847-F	TGATAAACTGTCTAACAAAACC	Construction of oriC plasmids for *M. feriruminatoris*
002	oriG5847-R	AATACTTACCTCCTTTACAAAAAAATAAC	
003	Pac-F	GGAAATATAAGATCCTTTGAATGACTGAATATAAACCTACTG	Exchange of *tetM* by *pac* marker
004	Pac-R	GGTTTTGTTAGACAGTTTATCATTAAGCACCAGGTTTTCTAG	
005	Pac_pS_as-F	GGTTTTGTTAGACAGTTTATCAGCAATTTATTTGGAAAATCTTTTTTTG	Creation of pIVB06
006	Pac_as-R	GTGAACAAGAAAACAGTGAATTAAGCACCAGGTTTTCTAG	
007	pIVB_pMFO-F	TGATAAACTGTCTAACAAAACC	
008	pIVB0X_as-R	TTCACTGTTTTCTTGTTCAC	
009	pIVB_pMFO3.1-F	CAAAAAAAGATTTTCCAAATAAATTGCGATTCATCAATATTTTTGTTAACC	Creation of pIVB08
010	pIVB_pMFO3.1-R	GGTTTTGTTAGACAGTTTATCAGGTTCAGAAACTGGTGCTTCAC	
011	pS'-F	GCAATTTATTTGGAAAATCTTTTTTTG	
012	TetM-R	CTAAGTTATTTTATTGAACATATATCGTAC	
013	oriC_right-F	GTACGATATATGTTCAATAAAATAACTTAGGGTGGTAGAGACCATACAAC	
014	oriC_right-R	GGTTCAGAAACTGGTGCTTCACTGATAAACTGTCTAACAAAACCATTC	
015	Puro-R	TTAAGCACCAGGTTTTCTAG	Creation of pIVB09
016	pIVB04.1-F	CTAGAAAACCTGGTGCTTAAGGTGGTAGAGACCATACAAC	
017	pIVB0X_scaff-F	GACGAAAGGGCCTCGTGATAC	Generation of plasmids carrying MIB/MIP gene copies under the control of their natural promoter regions
018	pIVB0X_scaff-R	TCGCGCGTTTCGGTGATG	
019	MM1_Mferi-F	CATCACCGAAACGCGCGAAAGTAGAAAAATTTTAATTAACATAAAATTTAG	
020	MM1_Mferi-R	GTATCACGAGGCCCTTTCGTCTTATTTGGCTTTATTATCTTTAAACTTG	
021	MM2_Mferi-F	CATCACCGAAACGCGCGATTTTAACTAAAATTTTAATATTGAATATATAAAAAAATAC	
022	MM2_Mferi-R	GTATCACGAGGCCCTTTCGTCTTATATAGTTTCTTTATTTTTAAATTCAAACTC	
023	MM3_Mferi-F	CATCACCGAAACGCGCGAGTAATTTATATTTTTTAGACAAAATGTGTTAAACC	
024	MM3_Mferi-R	GTATCACGAGGCCCTTTCGTCTTATTTGGCAGGGGTTCTAAATTTG	
025	MM4_Mferi-F	CATCACCGAAACGCGCGACTAAATTTGTGGTTTACTTGTAATTTTAAAATTATTTG	
026	MM4_Mferi-R	GTATCACGAGGCCCTTTCGTCTTATTTTTTGTTATCGAATTTAAACTCAC	
027	MM1_Mhyop-F	CATCACCGAAACGCGCGAGTTTTCTTATAATACTAAAAATTTGACTTTGC	
028	MM1_Mhyop-R	GTATCACGAGGCCCTTTCGTCTTATTTTCAGTCACCAAAAACATC	
029	MM2_Mhyop-F	CATCACCGAAACGCGCGAACAGGTAAAATTTATTTATTCTTTTAAAGTAG	
030	MM2_Mhyop-R	GTATCACGAGGCCCTTTCGTCTTAATTTGAATTTGAATTATTAAATTTATAATCGG	
031	MM_Mhyor-F	CATCACCGAAACGCGCGAAAGCTTTTATCTTTCTTTAGTTAAAACAAG	
032	MM_Mhyor-R	GTATCACGAGGCCCTTTCGTCCTAATTTACTACTTTTTTTTGTGTAGAAGG	
033	SCRpIVBX-F	ATGTGTCAGAGGTTTTCACC	Screening of plasmids in *E. coli* and in *M. feriruminatoris*
034	SCRpIVBX-R	CACCTGACGTCTAAGAAAC	
035	SCRMM1mfe-F	CATATAGAGAAGTGATGAAAG	
036	SCRMM1mfe-R	GCTAAACTAGCTATTAAAAC	
037	SCRMM2mfe-F	TGGTAAATCATATAGAGAAG	
038	SCRMM2mfe-R	GCTAAACTAGCTAATAAGGC	
039	SCRMM3mfe-F	GGAAAATCATATAGGGAAGC	
040	SCRMM3mfe-R	CCAAATGAAACTGAAGTAGC	
041	SCRMM4mfe-F	GGTAAATCCTATAGAGAAGC	
042	SCRMM4mfe-R	GATTCCATTTGATGATGTATC	
043	SCRMM1mhp-F	TGATGATAGCAACAAAGTTG	
044	SCRMM1mhp-R	ATTACAGCAACTGTAAGTCC	
045	SCRMM2mhp-F	TATGGTAGCGAATTTAAAAC	
046	SCRMM2mhp-R	GGGGATTAGCAAAAAAACTC	
047	SCRMMmhr-F	AGTAAATCAAATTCCTGACG	
048	SCRMMmhr-R	GGAACACTTAAGACAGTTAC	
049	SCRMM4mmc-F	ATGAATAAAATGTATAGCGG	
050	SCRMM4mmc-R	GAAGCAGCTAAACTAGCTAC	
051	scaff-pMM1mfe-R	TGTATTGCTCCTAAATAGAA	Generation of plasmids carrying MIB/MIP gene copies under the control of P_{MM1mfe} promoter
052	pMM1mfe_MM2mfe-F	TAAAATTCTATTTAGGAGCAATACA GTGTATTTTTTAAAAAAGAAGAAAAAATAAA	
053	pMM1mfe_MM3mfe-F	TAAAATTCTATTTAGGAGCAATACA ATGCATATATGAAAGAAGAAGAAAAATAAA	
054	pMM1mfe_MM4mfe-F	TAAAATTCTATTTAGGAGCAATACA GTGTATTTTTTAAGAAAGAAAAAAAAATAAA	
055	pMM1mfe_MM1+2mhp-F	TAAAATTCTATTTAGGAGCAATACA ATGCTGTTTTATTTATCGAAAAG	
056	pMM1mfe_MMmhr-F	TAAAATTCTATTTAGGAGCAATACA ATGAATTTTTTAAAGAAAACAAAAAAATTTC	
057	pMM1mmc-F	CATCACCGAAACGCGCGA CAGGAATTTCATTTGCTGC	Creation of a plasmid carrying the MIB/MIP gene copy #4 of *Mmc* GM12 6xHis and FLAG tagged
058	pMM1mmc:MM4mmc-R	GATTTTATTTTTTTTCTTTTTTAAAAAATACACTATTTTACTCCTTGAATAGAATTTCATAT G	
059	MIB4mmc-F	GTGTATTTTTTAAAAAAGAAAAAAAATAAAATC	
060	MIB4mmc_6His-R	CTTTTCATAGGTTATATATTCCTTTTTTTATTAATGATGATGGTGATGATGTGTAAATGATC TATCAGAAGC	
061	MIP4mmc-F	TAAAAAAAGGAATATATAACCTATGAAAAG	
062	MIP4mmc_FLAG-R	GTATCACGAGGCCCTTTCGTCTTATTTATCATCATCATCTTTATAATCGTTTTGTGTACCAT TATTGA	
071	SCRMM1mhpopt-F	ACGTATCTGTTTCCTAATGG	Screening of plasmids in *E. coli* and in *M. feriruminatoris* carrying codon-optimized MIB/MIP gene copies
072	SCRMM1mhpopt-R	TACAGCAACTGTTAATCCAC	
073	SCRMM2mhpopt-F	TGAAAGAAATTCCAGCTGAC	
074	SCRMM2mhpopt-R	AAAAATGACATACCAGCAGC	
075	SCRMMmhropt-F	GATATCACAACTAATACAAATCC	
076	SCRMMmhropt-R	AAGAGAATAGCACTTGTTGG	
077	RACE_Pmm1_a	ATTGTAATTTTAGTTTTTGATCTAC	5'RACE
078	RACE_Pmm2_a	CTCTCTTTGTACTTTGTTTAGG	
079	RACE_Pmm3_a	CTCCACTTATAACAATTGTTC	

080	RACE_Pmm4_a	CCTTCTTCTTGTTATCTTAGG	
081	RACE_Pmm1_b	GGTTTTGCTGCATCTTGTTG	
082	RACE_Pmm2_b	CATTAGGTTCTTTTTTATCTGC	
083	RACE_Pmm3_b	GGTATTACTAATCTTTCAGTAC	
084	RACE_Pmm4_b	TCTTCTTTTTTGGCTGGTGG	
085	Oligo dT-Anchor	GACCACGCGTATCGATGTCGACTTTTTTTTTTTTTTTTV	
086	RACE Anchor	GACCACGCGTATCGATGTCGAC	
087	5'RACE_pUC57anch-R	GTCGACATCGATACGCGTGGTCATCATTGGGACGTCAGGTG	Cloning and screening of 5'RACE fragments
088	5'RACE_pUC57mib1-F	CAACAAGATGCAGCAAAACCATCGGAAAGAACATGTGAGC	
089	5'RACE_pUC57mib2-F	GCAGATAAAAAAGAACCTAATGATCGGAAAGAACATGTGAGC	
090	5'RACE_pUC57mib3-F	GTACTGAAAGATTAGTAATACCATCGGAAAGAACATGTGAGC	
091	5'RACE_pUC57mib4-F	CCACCAGCCAAAAAAGAAGAATCGGAAAGAACATGTGAGC	
092	SCRpUC57m-F	ACATTTCCCCGAAAAGTGCC	
093	SCRpUC57m-R	TTGCTCACATGTTCTTTCCG	
094	Pmm2-R	TATTTTTCTCCTTAAAGATATAGATATTCTTTTG	Generate plasmids to test promoter activity
095	Pmm3-R	AGTTTCAACTCCTTTAAATATCAAAATAAC	
096	Pmm2_mKO2-F	TATCTTTAAGGAGAAAAATAATGGTTTCAGTTATTAAACCAG	
097	Pmm3_mKO2-F	TATTTAAAGGAGTTGAAACTATGGTTTCAGTTATTAAACCAG	
098	mKO2_pIVB0X-R	GTATCACGAGGCCCTTTCGTCTTATGAATGAGCAACAGC	
099	W/oPromotor-F	CCGAAACGCGCGAATGGTTTCAGTTATTAAACCAG	
100	ΔMIBMIPmfe-F	TTAAAATATATAAGATATAAGTGCATAAAATTCTATTTAGGAGCAATACA CGCTTGCCTGTAACTTACAC	Generate the ΔMIB/MIP strain by CReasPy-Cloning
101	ΔMIBMIPmfe-R	TTTAAAGAAATCATTTATTCTGTCTCCTTAGATATGTAGAAAGTTTTAATCTAAGTTATTTT ATTGAACATATATCGTACTTTATC	
102	gRNAΔMIBMIP-1-F	ATGATCTGATGAACTGTAAATCCAGGGT	
103	gRNAΔMIBMIP-1-R	TAAAACCCTGGATTTACAGTTCATCAGA	
104	gRNAΔMIBMIP-2-F	ATGATCGATTGATCAGGACTAAGCCCGT	
105	gRNAΔMIBMIP-2-R	TAAAACGGGCTTAGTCCTGATCAATCGA	
106	Mferi-F1B	TAATGTATGGAGCGTGTACTAC	MLST *M. feriruminatoris*
107	Mferi-R1B	TGGTATGGGAGATCATTGAATC	
108	Mferi-F2	GATGTGTTGATTATTGATGATG	
109	Mferi-R2	TCTTCTGGATTTTGTTGAGATC	
110	Mferi-F8	TACATACATCCAGCATCAATAC	
111	Mferi-R8	GTCAAGAATATATGAATGCTGG	
112	Mferi-F3	GTAGTTGCACTCATTTCATCATC	
113	Mferi-R3	GTTGGAGTTATTGGTCAAGCTG	
114	Mferi-F7	GAATGAAGTTGAAGTAGTTTTAG	
115	Mferi-R7	GTTGTACCTGGACGTAAATCG	
116	Mferi-F6	CAACTGAACCACGAGAACCAAC	
117	Mferi-R6	CTAGCTAGAACTAGTACATTAG	
118	Mferi-F4	CTTCAGCTAGTTGTTCAACAGC	
119	Mferi-R4	AGCAGGTATTGAAGCTTTAGTTG	
120	Mferi-F5	CATGCAACGTCAAGCTGTTCC	
121	Mferi-R5	CTAATTCCAGATTCTCCATCAG	

Table S2. List of plasmids used and created in this study

Plasmid	Genotype	Used in	Source
**p426_pgRNA-1MIBMIP**		CReasPy-Cloning	This work
**p426_pgRNA-2MIBMIP**		CReasPy-Cloning	This work
**pMT85-PSTetM-ARSCenHis-pRS313**		Generation of the recombination templates	(Labroussaa et al., 2016)
**pMYCO1**	$oriC_{Mmc}$ pS'*tetM*	Generate pIVB backbone	(Lartigue et al., 2003)
**pIVB03**	$oriC_{Mfe}$ pS'*tetM*	Transformation efficiency	This work
**pIVB04**	$oriC_{Mfe}$ pS'pac	Transformation efficiency	This work
**pIVB06**	$oriC_{Mfe}$ pS'pac	Transformation efficiency	This work
**pIVB08**	$oriC_{Mfe}$ pS'*tetM* Δ*dnaA*	Transformation efficiency	This work
**pIVB09**	$oriC_{Mfe}$ pS'*pac* Δ*dnaA*	Transformation efficiency	This work
**pIVB09_MM1mfe**	$oriC_{Mfe}$ pS'*pac* Δ*dnaA* P_{MM1mfe}MIB/MIP1_{mfe}	Expression of native MIB/MIP	This work
**pIVB09_MM2mfe**	$oriC_{Mfe}$ pS'*pac* Δ*dnaA* P_{MM2mfe}MIB/MIP2_{mfe}	Expression of native MIB/MIP	This work
**pIVB09_MM3mfe**	$oriC_{Mfe}$ pS'*pac* Δ*dnaA* P_{MM3mfe}MIB/MIP3_{mfe}	Expression of native MIB/MIP	This work
**pIVB09_MM4mfe**	$oriC_{Mfe}$ pS'*pac* Δ*dnaA* P_{MM4mfe}MIB/MIP4_{mfe}	Expression of native MIB/MIP	This work
**pIVB09_P_{MM2mfe}:mKO2**	$oriC_{Mfe}$ pS'*pac* Δ*dnaA* P_{MM2mfe}*mKO2*	Testing of promoter activity	This work
**pIVB09_P_{MM3mfe}:mKO2**	$oriC_{Mfe}$ pS'*pac* Δ*dnaA* P_{MM3mfe}*mKO2*	Testing of promoter activity	This work
**pIVB09_Ø:mKO2**	$oriC_{Mfe}$ pS'*pac* Δ*dnaA mKO2*	Testing of promoter activity	This work
**pIVB09_ P_{MM1mfe}:MM2mfe**	$oriC_{Mfe}$ pS'*pac* Δ*dnaA* P_{MM1mfe}MIB/MIP2_{mfe}	Expression of native MIB/MIP	This work
**pIVB09_ P_{MM1mfe}:MM3mfe**	$oriC_{Mfe}$ pS'*pac* Δ*dnaA* P_{MM1mfe}MIB/MIP3_{mfe}	Expression of native MIB/MIP	This work
**pIVB09_MM1mhp**	$oriC_{Mfe}$ pS'*pac* Δ*dnaA* P_{MM1mhp}MIB/MIP1_{mhp}	Expression of foreign MIB/MIP	This work
**pIVB09_MM2mhp**	$oriC_{Mfe}$ pS'*pac* Δ*dnaA* P_{MM2mhp}MIB/MIP2_{mhp}	Expression of foreign MIB/MIP	This work
**pIVB09_MMmhr**	$oriC_{Mfe}$ pS'*pac* Δ*dnaA* P_{MMmhr}MIB/MIP_{mhr}	Expression of foreign MIB/MIP	This work
**pIVB09_ P_{MM1mfe}:MM1mhp**	$oriC_{Mfe}$ pS'*pac* Δ*dnaA* P_{MM1mfe}MIB/MIP1_{mhp}	Expression of foreign MIB/MIP	This work
**pIVB09_ P_{MM1mfe}:MM2mhp**	$oriC_{Mfe}$ pS'*pac* Δ*dnaA* P_{MM1mfe}MIB/MIP2_{mhp}	Expression of foreign MIB/MIP	This work
**pIVB09_ P_{MM1mfe}:MMmhr**	$oriC_{Mfe}$ pS'*pac* Δ*dnaA* P_{MM1mfe}MIB/MIP_{mhr}	Expression of foreign MIB/MIP	This work
**pIVB09_ P_{MM1mmc}:MM4mmc**	$oriC_{Mfe}$ pS'*pac* Δ*dnaA* P_{MM1mmc}MIB/MIP4_{mmc}	Expression of foreign MIB/MIP	This work
**pIVB09_ P_{MM1mfe}:MM4mfe 6xHis/FLAG**	$oriC_{Mfe}$ pS'*pac* Δ*dnaA* P_{MM1mfe}MIB/MIP4_{mfe} 6xHis/FLAG	Expression of foreign MIB/MIP	This work
**pIVB09_ P_{MM1mmc}:MM4mmc 6xHis/FLAG**	$oriC_{Mfe}$ pS'*pac* Δ*dnaA* P_{MM1mmc}MIB/MIP4_{mmc} 6xHis/FLAG	Expression of foreign MIB/MIP	This work
**pIVB09_ P_{MM1mfe}:MMmhr 6xHis/FLAG**	$oriC_{Mfe}$ pS'*pac* Δ*dnaA* P_{MM1mfe}MIB/MIP_{mhr} 6xHis/FLAG	Expression of foreign MIB/MIP	This work
**pIVB09_ ds:MMmhr 6xHis/FLAG**	$oriC_{Mfe}$ pS'*pac* Δ*dnaA* P_{MM1mfe}dsMIB/MIP_{mhr} 6xHis/FLAG	Expression of chimeric foreign MIB/MIP	This work
**pIVB09_MIB4mfe**	$oriC_{Mfe}$ pS'*pac* Δ*dnaA* $P_{MM4mfe}$$MIB_{mfe}$	Expression of single MIB/MIP partners	This work
**pIVB09_MIP4mfe**	$oriC_{Mfe}$ pS'*pac* Δ*dnaA* P_{MM4mfe}MIP4_{mfe}	Expression of single MIB/MIP partners	This work

Table S3. List of antibodies used in this work

Antigen	Target species	Host/Source	Clonality	Conjugation	Dilution in WB	Reference	Supplier
**IgG (H+L)**	Goat	Mouse	Polyclonal	-	1:1000	205-005-108	Jackson Immunoresearch
**IgG (H+L)**	Pig	Rabbit	Polyclonal	-	1:5000	P0916-2ML	Merck
**Polyhistidine**	-	Mouse	Monoclonal	-	1:2000	H1029	Merck
**FLAG peptide**	-	Mouse	Monoclonal	-	1:2000	F1804	Merck
**mKO2**	-	Rabbit	Polyclonal	-	1:2000	M168-3M	MBL
***E. coli* DnaK**	-	Rabbit	Polyclonal	-	1:2000	PA5-117658	ThermoFisher
**IgG**	Mouse	Rabbit	Polyclonal	HRP	1:2000	P0260	Dako
**IgG**	Rabbit	Donkey	Polyclonal	HRP	1:10.000	31458	ThermoFisher

Supp. Figure S1. Analysis of MIB-MIP gene pair expression in *Mmc* GM12 using -omics. A) Transcriptional analysis using RNAseq of three biological replicates. Top graph shows the mean fragments per kilobase of transcript per million mapped reads (FPKM) of each gene of the MIB-MIP cluster. Bottom graph indicates the read coverage obtained in each replicate. **B)** Total gene distribution based on gene expression measured by RNAseq. **C)** Total protein distribution based on protein expression measured by mass spectrometry.

Supplementary Figure S1

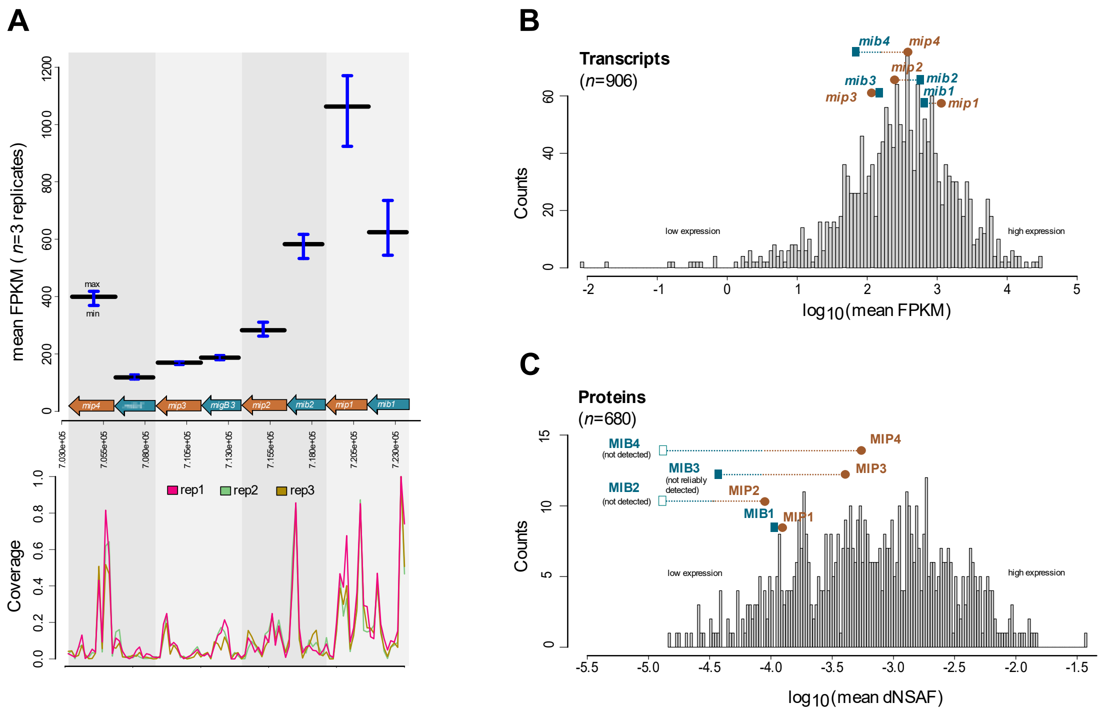

Supp. Figure S2. 5'RACE fragment analysis by agarose gel electrophoresis. Lane MW, GeneRuler 1 kb DNA ladder; Lanes 1-4, 5'RACE results derived from primers targeting MIB1 to MIB4, respectively. Note the double band present in lane 2 (MIB2).

Supplementary Figure S2

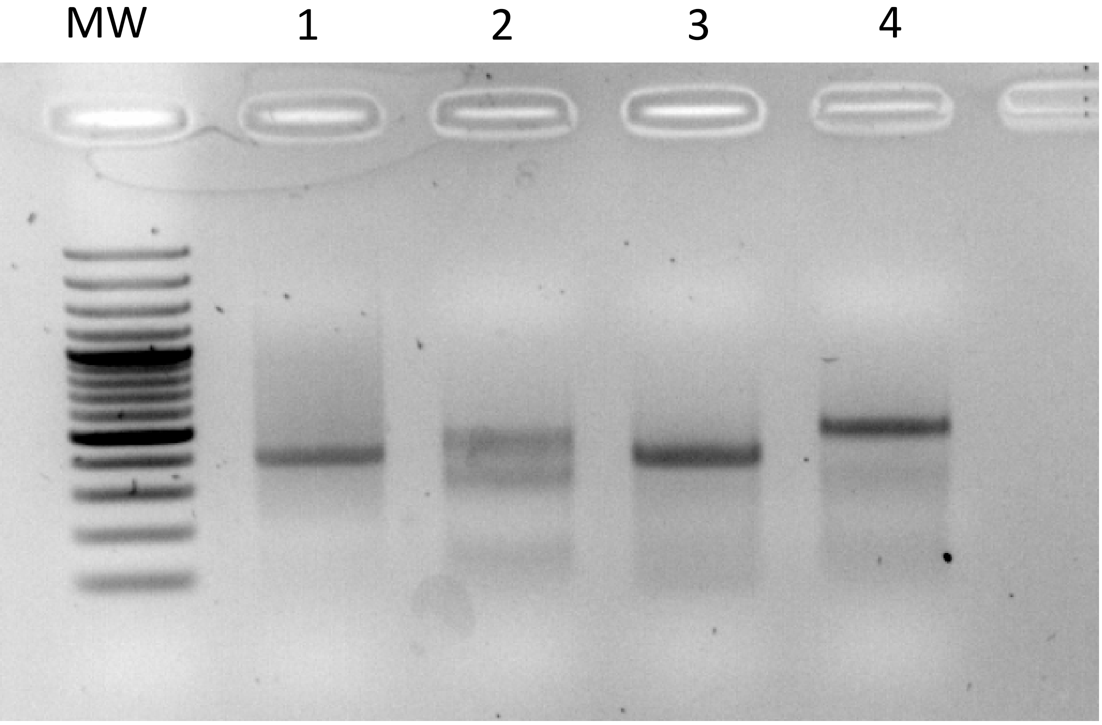

Supp. Figure S3. A) Schematic representation of the procedure followed to obtain the *Mferi* ΔMIB-MIP strain. Briefly, the genome of *Mferi* IVB14/OD_0535 wild-type was isolated and transferred and modified in *S. cerevisiae* in a single step using CReasPy cloning, as described in the Materials and Methods section. Then, modified genomes were isolated and transplanted into an *Mcap* ΔRE strain to obtain the final mutant. B) Multi-locus Sequence Typing of several clones obtained after transplantation. Only clones 1, 5 and 6 contained the modified *Mferi* genome. + indicates the positive control.

Supplementary Figure S3

A

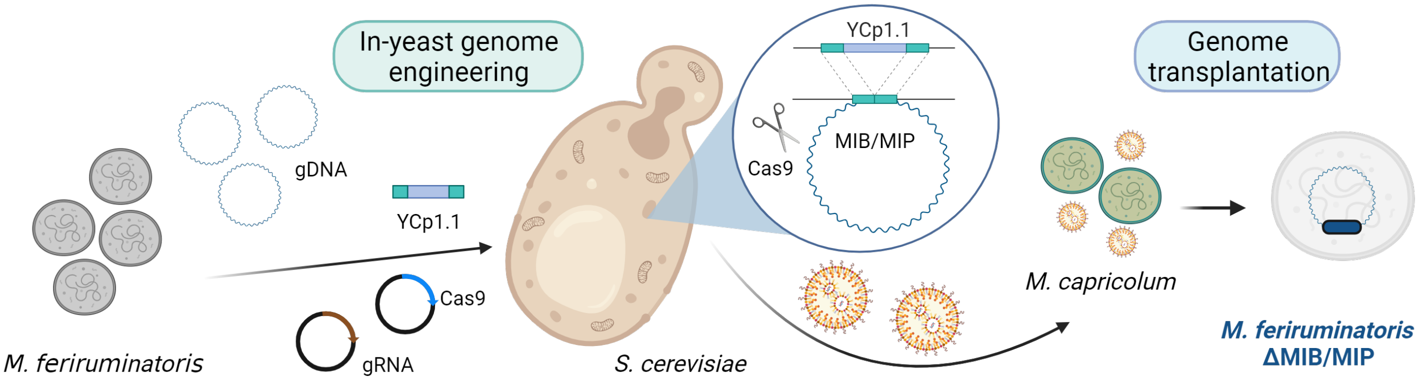

B

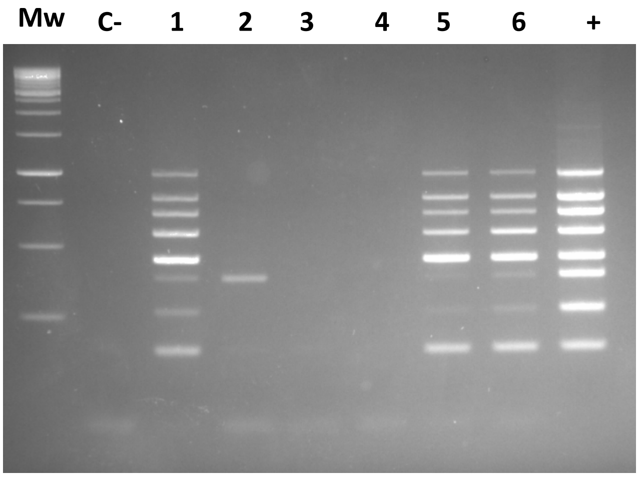

Supp. Figure S4. Determination of pIVB09 stability and loss upon serial passaging without antibiotic pressure (green) and with 8 µg mL^{-1} (blue) or 16 µg mL^{-1} (black) of puromycin. Graphic displays the results of three independent biological replicates.

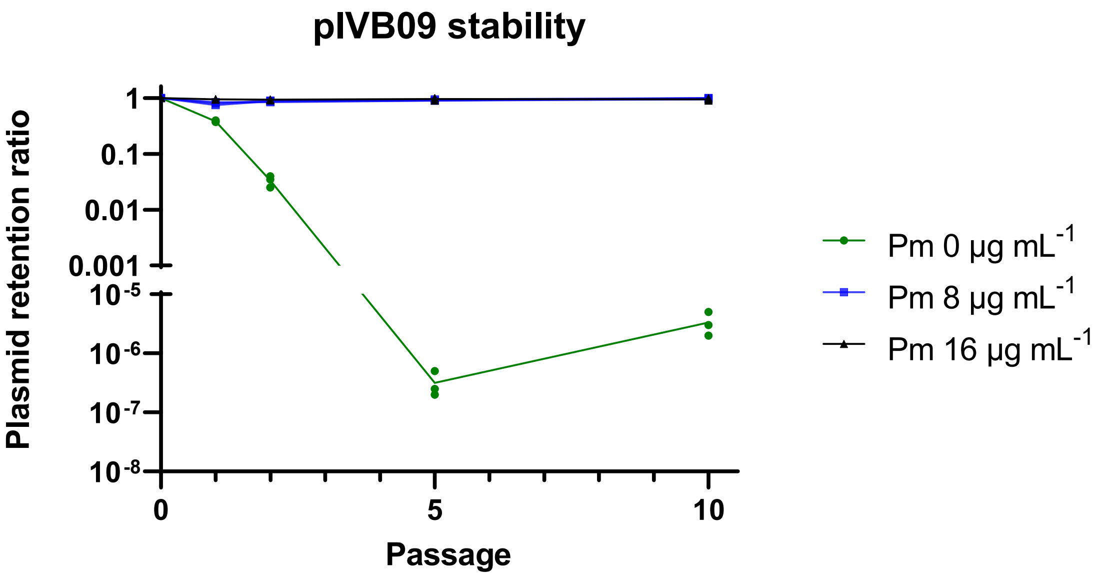
pIVB09 stability
Plasmid retention ratio
1
0.1
0.01
0.001
10-5
10-6
10-7
10-8
0
5
10
Passage
Pm 0 µg mL-1
Pm 8 µg mL-1
Pm 16 µg mL-1

Supp. Figure S5. Analysis of promoter activity of the upstream regions of MIB2 and MIB3 of *Mferi*. A) mKO2 protein expression analysis of *Mferi* ΔMIB-MIP mutant carrying the pIVB09 plasmid with transcriptional fusions of the mKO2 gene with the MIB2, MIB3 or no promoter at all (P_{MM2}, P_{MM3} or Ø). B) Detection of presence of mKO2 in colonies of the respective mutants studied in A). First row shows the brightfield image, second row the TRITC channel and bottom row the merge of the previous two.

Supplementary Figure S5

A

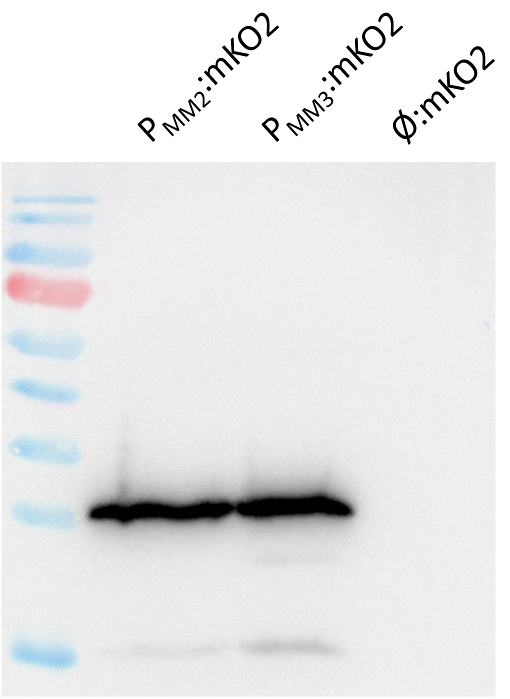

B

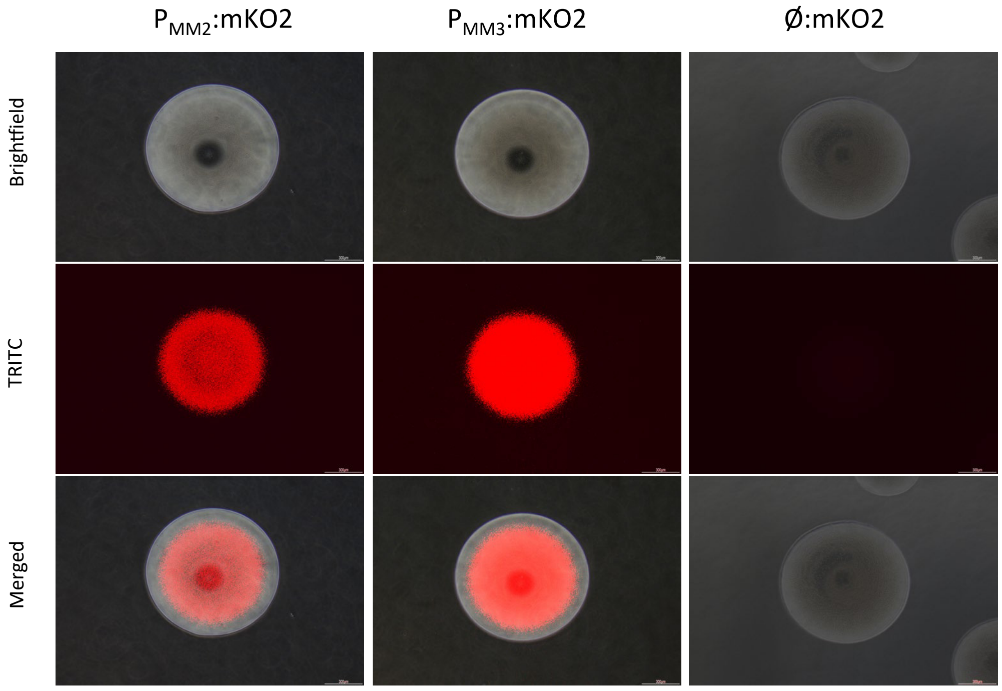

Supp. Figure S6. A) Representation of the location of the different MIB-MIP related genes in the chromosome of *M. hyopneumoniae* Ue273. The two complete MIB-MIP pairs are located in close proximity, while there is also at least one gene with homology to MIB with no following MIP partner. The MIB-MIP related ATPase gene cluster is not located immediately after the MIB-MIP operon, as it is the case in almost all species analyzed. B) Upstream DNA sequence of the first gene of the MIB-MIP related ATPase gene cluster. Putative Shine-Delgarno, Pribnow box and -35 sequence are underlined or highlighted in yellow or green, respectively. -16 (AG) and -35 sequences are separated by a sequence of consecutive adenines, typically found in mycoplasma promoters with DNA slippage control.

Supplementary Figure S6

A

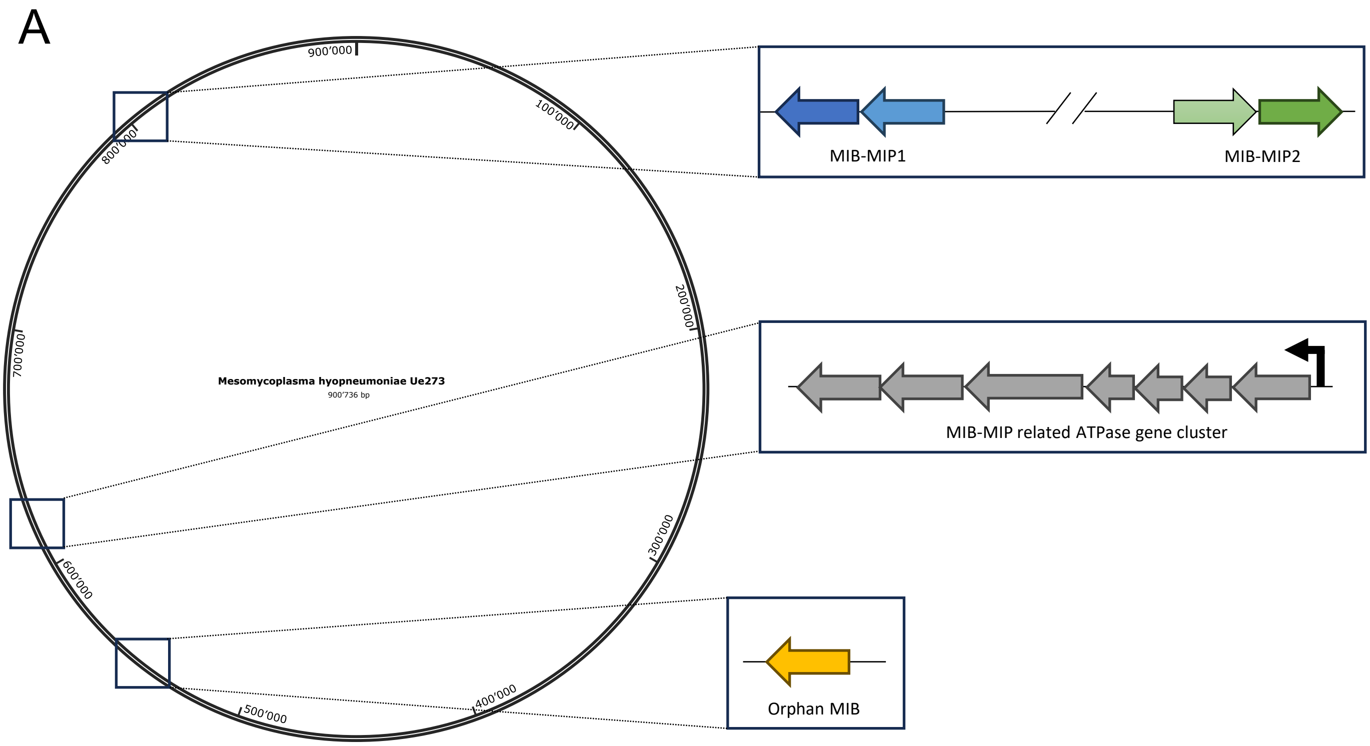

B

TTCATTTTTTTGGCAAAAAAAAAAAAAAAGTTATAATATATTTC
ATCTTTGAAAAAACTAGGAAAGGAGAAATA**ATG**$_{614593}$

Supp. Figure S7. Sequence alignment of the N-terminal residues of the MIB (A) and MIP (B) proteins of *M. hyorhinis* JF5820, *Mferi* IVB14/OD_0535 (4th pair) and *Mmc* GM12 (4th pair).

Supplementary Figure S7

A

```
MIBmhr      MNFLKKTKKFLKKSKNQQVLFSFVTVLSVPSVVG-IIYYVASKNTDSKEDVKILDKLNAN     59
MIB4mfe     -------VYFLRKKKNKILVLALVSSLATSASFGSVIYYSFSDANISFDT-SSN---GIT     49
MIB4mmc     -------VYFLKKKKNKILTIALVASLAASVSFGSVLYYSFSDNHISFDT-SSN---GIT     49
                     **:*.**: : :::*: *:.   .* ::**  *.    * :  .     . .

MIBmhr      SVTLQSIENADTNKALDALNDKNLKPNPKPEEKKPDPVPTPPVVVNPPKTPPKTPKPEPA     119
MIB4mfe     DAELAPINNA-ADDAIVSNRDNKLKPSPERIIKEAEKKIEEKIIIPPAKKEEKIEAAKPI     108
MIB4mmc     DAELAPINNA-INDAIVSNRDNKLKPSEEKIIKETEKKIEEKIIIPPAKKEEKIEAAKPI     108
            .. *  *:**  :.*: : .*::***. :   *: :      ::: * *.  *    :*

MIBmhr      PEP--ETPPVVEPAPSIQQTFKDDETGYVTLKWEGHDVRALVRKKPNRQYSTYDNQNGLA     177
MIB4mfe     PKPVVKKPDAPLSSPKITRR-------RQKITISGIEVEAEIEGPPSFVVHKRDKDRGIA     161
MIB4mmc     PKPVVRKPETKITSPKITRR-------KQTITIAGIEVEAEIEGPPGFVTHQRDKDRKIS     161
            *:*  ..* .   :*.* :          .:.  * :*.* :.  *.      *::. ::

MIBmhr      -NRIPYQSQVIDEIINIEVTPEIQAQNLKNTATALKNSVQGSAFSIYADTISQGDKAVAA     236
MIB4mfe     NPTRPYQNHTVNKILNVTVTKELKEKVAQDALTGGTGYDEG-------------------     202
MIB4mmc     NPTKPYQNHTVNKILSVKVTDKLKEQVAKDALSGGNGYDEG-------------------     202
                ***.:.:::*:.: ** ::: :  ::: :. ..  :*

MIBmhr      VVAQNYENYYKNMFSKWQQLFDNGDKVREFLTKEGYDLYPTIKQTYENDLKKAKEAIEKA     296
MIB4mfe     -----AGLFNNSLFNVFKAEFDSSKKLDDILSSLEAV-AHQNSGTFQNTLERYKKLL---     253
MIB4mmc     -----VGLFNNSIFNVFKEEFNSGKELNDILSSLESV-ARQNSGAFQNTLERYKKML---     253
                    : :.:*. ::  *:...:: ::*:.         . :::* *:: *: :
```

B

```
MIPmhr      MKKNRKSRNLLLKNLLISSAFIMPIL-VVSCSDTSNNPAKSETSSQSSDKTNPDTSDPNT     59
MIP4mfe     MKR----LNKLL-MYASSSTLLLPITLLVACTPSKVV-SRPID----DNVFNK-LINSIK     49
MIP4mmc     MKR----LNKLL-MYISSSTLLLPITLLVACTPSKVV-AKPIN----DDEFNK-LIDSIK     49
            **:     * **     **::::**  :*:*: :.   ::        .:  *     :  .

MIPmhr      TTPSILFNADK---TPKEITTINLEKLPIILKRVDHFSNYSNLSTNSINTSLADLNKQLE     116
MIP4mfe     TEEDLLKYADIKFKDQRGSETSKGNILPSQLKKEDVTITFKG------------------     91
MIP4mmc     TENDLLKYADIRFKNPNGADTKKEDIIPSQLKSENISITFKG------------------     91
            *   .:*  **       .    * : : :*  **   :    .:..

MIPmhr      FATTNKDDNDQIQIQVENKKYLNNNPETSLEQGKLIFSL---KISSKKNPNISITKDVEV     173
MIP4mfe     ------KYSGQIFTEVLNVNVINQD-SSSRNKVNIFVQFTNKKTGTKIPISFVVSGLNEN     144
MIP4mmc     ------KYQGQISAVVTNVDVDRTNPFAVQNEATIFVQFKNLKTNTSKPISITIKGLNQK     145
                  . ..**   * * .  . :  :  :: .::..:   * .:.   .: :.   :

MIPmhr      GGFRQSRSSEINLNWANRFAPGSEDEFKQYFAENNYQRYETDAKKYFQALNGQLSLIEGK     233
MIP4mfe     GDFDFSGTRIIN----DLDYFGGLSGFNDYSSKTQEQRFDYDNLRYTTGLKNHLSSGTG-     199
MIP4mmc     GNFDASGNIVVD----DFAYFGGTSGYDEYTKKDQKSRFDYDNERYMTRLKSQFGNSSN-     200
            *.*  * .  ::     :    *. . :.:*  : : .*:: *  :*   *:.::.   .

MIPmhr      SPFDINSWRPDVKRTPEEVKKYDELAKQLKLDTYDDALKKGFTMPVYDDKGNVEGLKLYD     293
MIP4mfe     -TVDLRKLR-GLDTKEEQIRTFDKLAKEVKFDSYYNAALKGFTLPVYDSSGKFKGLSVND     257
MIP4mmc     -SINLKEYR-GLETKQENIKKFDDQAAISNFDTYYNAALKGFTLPVYGSDGKVSGLKIYE     258
              .::.. * .:. . *:::.:*. *   ::*:* :*  ****:***...*:..**.: :
```

Supp. Figure S8. Assessment of goat IgG cleavage activity of mutants only expressing the MIB or MIP protein in the *Mferi* ΔMIB-MIP strain. 1) WT strain, 2) goat IgG control, 3) ΔMIB-MIP + pIVB09_MIB1mfe and 4) ΔMIB-MIP + pIVB09_MIP1mfe.

Supplementary Figure S8

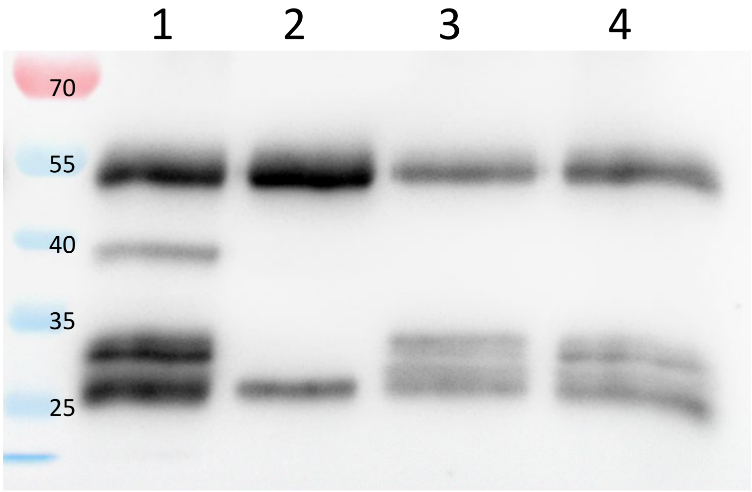

Supp. Figure S9. Longitudinal evaluation of clinical symptoms (**A-D**) and hematologic parameters (**E-H**) over the course of *Mferi* or *Mccp* infection. (**E-H**) Counts in the peripheral blood of white blood cells (WBC) (**E**), lymphocytes (**F**), monocytes (**G**) and neutrophils (**H**) over the course of disease. Data were obtained with VETSCAN HM5 Hematology Analyzer (Abaxis) and graphs were generated with the GraphPad Prism 9.4.0 software. Each symbol represents an individual animal (goats infected with *Mferi* IVB14/OD_0535, n = 3, in green; goats infected with *Mccp* ILRI181, n = 3, in red).

Supplementary Figure S9

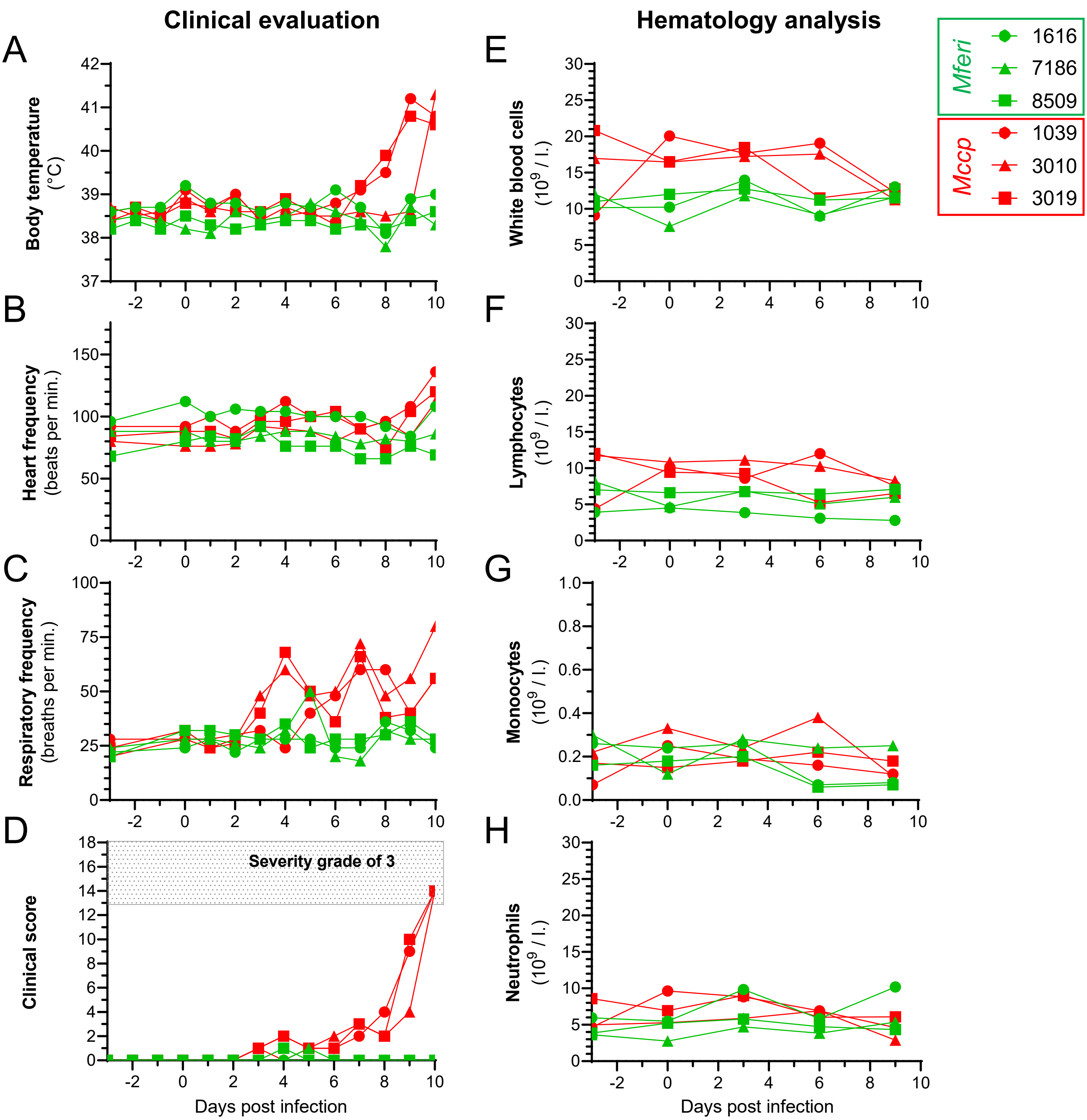

Supp. Figure S10. A) Sequence alignment of the first 60 residues of all the MIB and MIP proteins of *Mferi* IVB14/OD_0535 and *Mmc* GM12. The Cysteine involved in the anchoring of the MIP to the membrane is indicated with an arrow. B) Signal peptide prediction of the first MIB-MIP pair of *Mferi* IVB14/OD_0535 by using SignalP 6.0. Top and bottom graphs show the results obtained with the MIB and MIP, respectively. C) Detection of 6xHis-tagged and FLAG-tagged MIB and MIP, respectively, in protein fractions obtained by Triton X-114 enrichment. DnaK is shown as a loading control.

Supplementary Figure S10

A

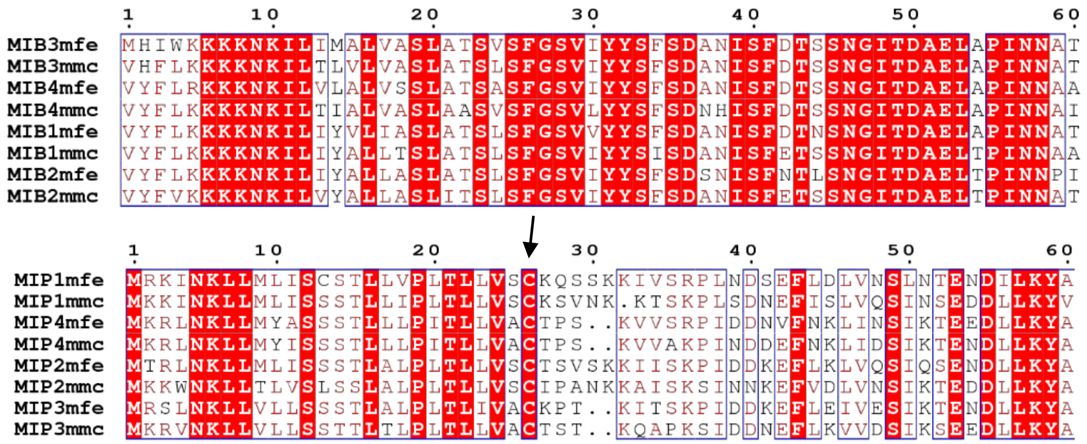

B

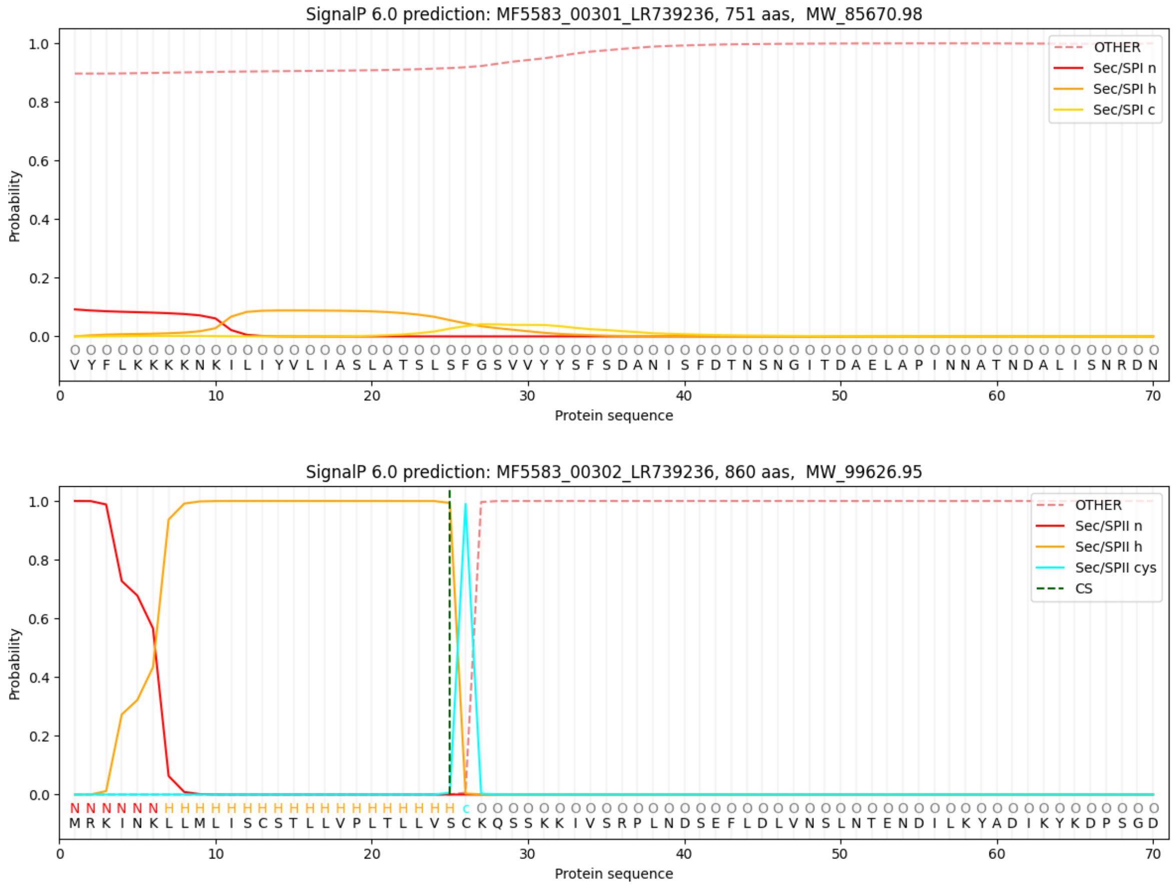

C

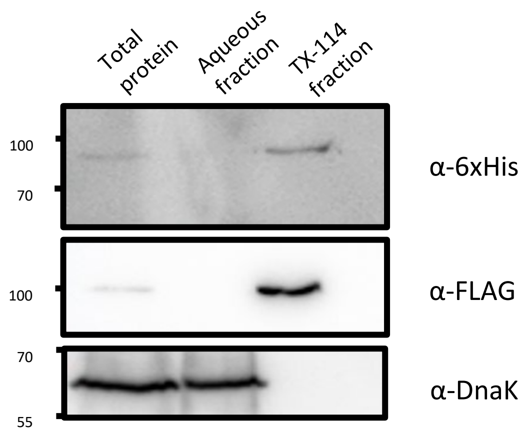

Supp. Figure S11. Uncropped western-blot images. A) Blot corresponding to Figure 5C and 5D. **B)** Blot corresponding to Figure 6B. **C) and D)** Blot corresponding to Figure 6C. **E)** Blot corresponding to Figure 7A (α-6xHis). **F)** Blot corresponding to Figure 7A and 7B (α-FLAG). **G)** Blot corresponding to Figure 7B (α-6xHis). **H)** Blot corresponding to Figure 7A and 7B (α-DnaK).

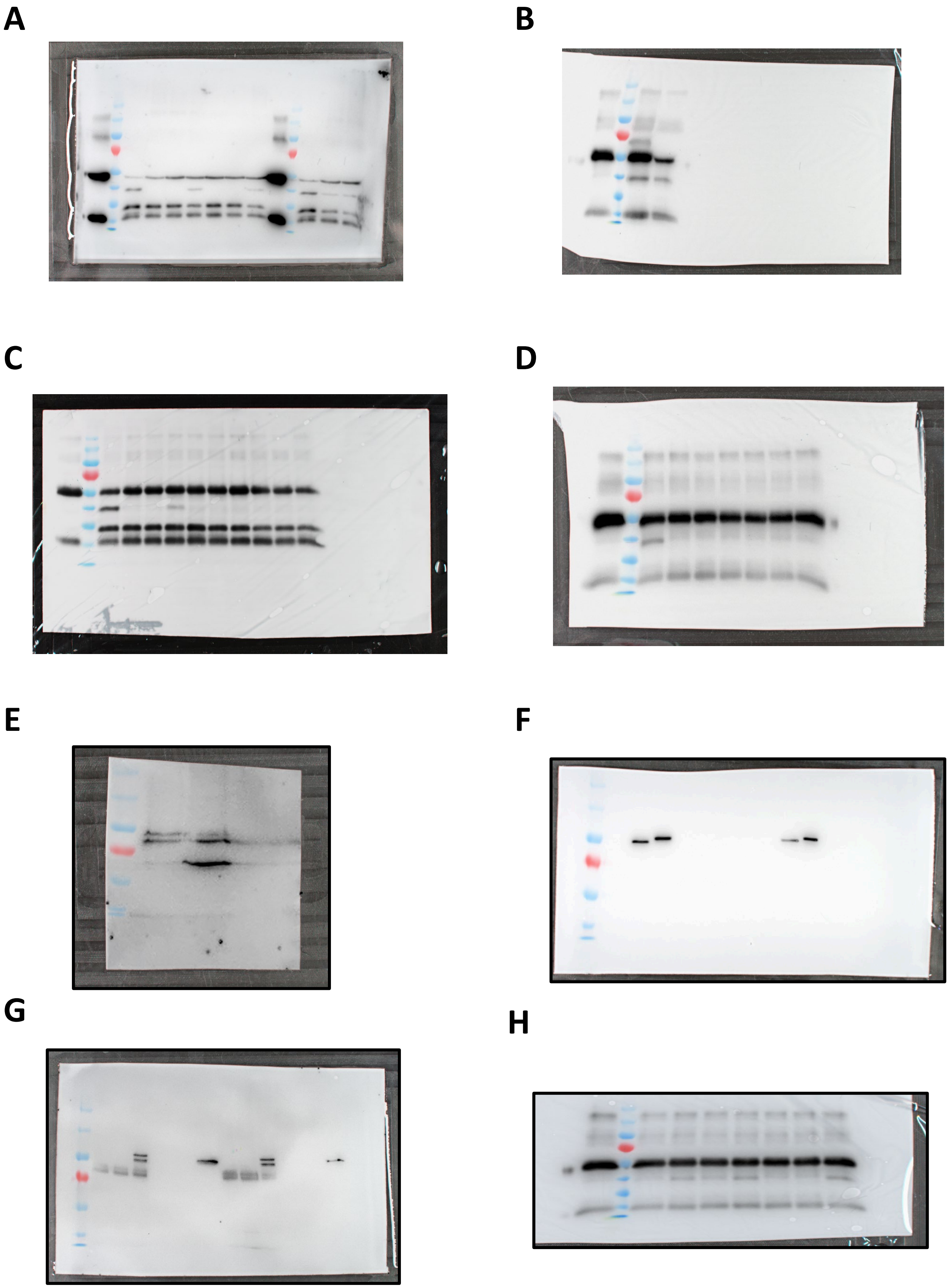
A
B
C
D
E
F
G
H
