# Supplementary material for: Functional surface expression of immunoglobulin cleavage systems in a candidate Mycoplasma vaccine chassis
Source: Commun Biol. 2024 Jun 28;7:779. doi: 10.1038/s42003-024-06497-8 (PMC11213901; doi:10.1038/s42003-024-06497-8)
Supplement: Supplementary file 2 — Description of Additional Supplementary Files [file 42003_2024_6497_MOESM2_ESM.pdf]

Description of Additional Supplementary Files

File name: Supplementary Data 1
Description: List of strains to build phylogenetic trees.

File name: Supplementary Data 2
Description: Raw Proteomics Data

File name: Supplementary Data 3
Description: Plasmid sequences in genbank format.
